# Quantification of Myocyte Disarray in Human Cardiac Tissue

**DOI:** 10.3389/fphys.2021.750364

**Published:** 2021-11-16

**Authors:** Francesco Giardini, Erica Lazzeri, Giulia Vitale, Cecilia Ferrantini, Irene Costantini, Francesco S. Pavone, Corrado Poggesi, Leonardo Bocchi, Leonardo Sacconi

**Affiliations:** ^1^Laboratory of Non-Linear Spectroscopy (LENS), University of Florence, Sesto Fiorentino, Italy; ^2^Division of Physiology, Department of Clinical and Experimental Medicine, University of Florence, Florence, Italy; ^3^National Institute of Optics, National Research Council, University of Florence, Florence, Italy; ^4^Department of Biology, University of Florence, Florence, Italy; ^5^Department of Physics, University of Florence, Florence, Italy; ^6^Department of Information Engineering, University of Florence, Florence, Italy; ^7^Faculty of Medicine, Institute for Experimental Cardiovascular Medicine, University of Freiburg, Freiburg im Breisgau, Germany

**Keywords:** 3D cardiomyocyte orientation, 3D FFT, cytoarchitecture reconstruction, disarray quantification, tissue modeling

## Abstract

Proper three-dimensional (3D)-cardiomyocyte orientation is important for an effective tension production in cardiac muscle. Cardiac diseases can cause severe remodeling processes in the heart, such as cellular misalignment, that can affect both the electrical and mechanical functions of the organ. To date, a proven methodology to map and quantify myocytes disarray in massive samples is missing. In this study, we present an experimental pipeline to reconstruct and analyze the 3D cardiomyocyte architecture in massive samples. We employed tissue clearing, staining, and advanced microscopy techniques to detect sarcomeres in relatively large human myocardial strips with micrometric resolution. Z-bands periodicity was exploited in a frequency analysis approach to extract the 3D myofilament orientation, providing an orientation map used to characterize the tissue organization at different spatial scales. As a proof-of-principle, we applied the proposed method to healthy and pathologically remodeled human cardiac tissue strips. Preliminary results suggest the reliability of the method: strips from a healthy donor are characterized by a well-organized tissue, where the local disarray is log-normally distributed and slightly depends on the spatial scale of analysis; on the contrary, pathological strips show pronounced tissue disorganization, characterized by local disarray significantly dependent on the spatial scale of analysis. A virtual sample generator is developed to link this multi-scale disarray analysis with the underlying cellular architecture. This approach allowed us to quantitatively assess tissue organization in terms of 3D myocyte angular dispersion and may pave the way for developing novel predictive models based on structural data at cellular resolution.

## 1. Introduction

Cellular orientation plays a key role in the electromechanical function of cardiac tissue. Myocyte geometrical features are determinant to define the direction of force production and the propagation pathways of the electrical signal, i.e., the action potential (Katz, 2010). At the same time, tension generation during the myocyte excitation depends on a proper sarcomeres spatial organization(Bers, 2001). Many heart diseases lead to remodeling processes that can occur at the sub-cellular level ( Røe et al., 2015; Heijman et al., 2016; Coppini et al., 2018; Denham et al., 2018; Scardigli et al., 2018) and in tissue architecture including myocardial disarray (Spinale and Zile, 2013; Hansen et al., 2017; Ariga et al., 2019). Cellular disarray is a complex process involving tissue organization at different scales, and it is heterogeneously distributed across the heart (Tseng et al., 2006; Bernus et al., 2015; GarciaCanadilla et al., 2019; Campanale et al., 2020).

Consolidated methods for obtaining an overall reconstruction of the fiber distribution in the heart (where the term “fibers” refer to the prevailing cardiomyocyte direction) generally employ DT-MRI and Phase-Contrast micro-CT (Varray et al., 2013, 2017; Nielles-Vallespin et al., 2017; NiellesVallespin et al., 2020; Reichardt et al., 2020) combined with modern image processing techniques such as 3D Fast Fourier Transform (3D-FFT) or Structure Tensor Analysis (STA) to estimate the local orientation of the fibers. These approaches can provide an overview of the heart organization, but their spatial resolution is insufficient to investigate the impact of structural remodeling at the single-cell level. Moreover, collagen deposition or different biological structures as nerves or vessels may impact the cytoarchitecture analysis (Greiner et al., 2018). Recently, Phase-Contrast micro-CT was combined with high-resolution imaging techniques such as Serial-Block-Face Scanning Electron Microscopy (SBF-SEM) or Focused Ion Beam-Scanning Electron Microscopy (FIB-SEM) to perform multi-scale investigations on the heart organization (Rykiel et al., 2020), and accurate analysis of the 3D organization of contractile units (Pinali et al., 2015; Willingham et al., 2020), although limiting 3D reconstruction with nanometric resolution at micrometers-sized volumes.

Optical methods can potentially overcome these limitations in terms of specificity, sample size, and spatial resolution. However, light scattering and absorption commonly make these methods not effective in massive samples. Recently, producing large, transparent, and fluorescent-labeled volumes has been made possible by applying a true tissue transformation approach (Chung et al., 2013; Tomer et al., 2014; Costantini et al., 2015; Pianca et al., 2019; Di Bona et al., 2020; Olianti et al., 2021). Coupling these methods with novel microscopy techniques enables to reconstruct specific biological structures with micrometric resolution in massive samples. Also, staining specific biological structures, such as intercalated disks, sarcomere, or cellular membranes, may provide useful information to detect the tissue organization in terms of cellular orientation in three dimensions.

In addition, analyzing the big amount of data needed to fully characterize the 3D cytoarchitecture at cellular level requires advanced tools of image analysis. Experimental studies (Feinberg et al., 2012; Drew et al., 2015, 2016; Pasqualin et al., 2016; Toepfer et al., 2019; Morris et al., 2020) performed on either isolated cells, monolayer engineered muscle tissues or histological samples, have provided new geometrical characterizations of inter- and intra-cellular architecture using two-dimensional (2D) image analysis techniques such as FFT or STA. These approaches are limited by the use of 2D images to directly estimate the 3D structural information. This issue was addressed in Bub et al. (2010), Botcherby et al. (2013), and Bensley et al. (2016), by extracting the 3D orientation of different biological structures through a complex 2D image elaboration. However, the used techniques are often based on manual selections or geometrical models. Recently adopted high-resolution imaging techniques as the SBF-SEM provide a sufficient spatial resolution to apply more accurate automatic image analysis (Hussain et al., 2018). These investigations provide new insights into the organization of myofibrils in cardiomyocytes, highlighting the importance of sarcomere arrangement, but are limited to the single cell level.

To date, an integrated and fully automated method for quantifying the tissue remodeling in terms of myocyte 3D angular dispersion at different scales in massive samples is missing.

In this study, we present a new experimental methodology to reconstruct and analyze the 3D myocyte angular dispersion on human cardiac muscle tissue strips. In the samples, the α-actinin protein was stained with a fluorescent probe to highlight Z-bands, exploited to detect the local longitudinal axis of myocytes at micrometric resolution. A two-photon fluorescence microscope (TPFM) was used to perform mesoscale reconstructions of the entire samples with sub-micron resolution. Then, we developed an automatic software pipeline able to equalize the images, segment the contractile tissue, and evaluate the 3D myocyte orientation along the entire sample by means of a 3D FFT-based approach. A 3D frequency filter was developed to select and quantify the regularity degree of the sarcomeric structures, preventing non-specific signals from corrupting the orientation analysis. The resulting virtual cytoarchitecture was stored as a vector map, and a metric was defined to quantify the 3D local tissue disarray with different spatial resolutions. The software collects the local disarray across the sample and visualizes its distribution with a 3D map. Finally, a virtual sample generator tool is proposed to simulate cardiac tissue with different cellular organizations to quantitatively interpret our data in terms of the 3D myocyte angular dispersion.

First, we tested the accuracy of the proposed FFT-based orientation analysis on a set of 140 two-photon reconstructions of human myocyte portions. Later, as a proof of concept, the proposed investigation was applied to two distinct classes of human cardiac tissues where the largest difference in the degree of fibers regularity is expected: InterVentricular Septum of a healthy donor (IVS) and atrial tissue provided by a patient suffering from chronic Atrial Fibrillation (AF). In fact, while in healthy septal myocardium the cells are packed (almost to constitute a crystalline structure) and the degree of orientation is very high, and in atria the regularity of the cell orientation is lower, especially in a pathological condition, where the extent of extracellular matrix and the cell misalignment are further worsened (Schotten, 2002; Schotten et al., 2003; Burstein and Nattel, 2008). The local disarray of IVS and AF tissue strips dissected congruently with fascicles in the cardiac wall was analyzed and characterized at different spatial resolutions to investigate the dependency between the tissue disarray and the spatial scale of analysis, and to prove the sensitivity of the proposed method. Finally, an example of the virtual sample generator scope is shown. A set of virtual samples were generated by real data with increasing levels of myocyte angular dispersion and analyzed with the proposed software pipeline. The virtual samples that best fit the preliminary results of the local disarray distributions found in human IV and AF tissues were used to predict the actual 3D angular dispersion of myocytes in the human samples.

## 2. Methods

### 2.1. Sample Preparation and Imaging

Three tissue strips (IVS1, IVS2, and IVS3) were collected from an interventricular septal sample of a healthy human donor (IVS), and two strips (AF1 and AF2) were collected from an atrial tissue sample of one patient with chronic Atrial Fibrillation (AF). Protocols for tissue collection and use were approved by the ethical committee of Careggi University-Hospital (2006/0024713; renewed May 2009). All strips (here also called multi-cellular preparations) were excised to a similar shape (about 1.5 mm in length and 0.4 mm in section), maintaining the preferential cardiac fibers direction along the main strip axis. Strips were demembranized by means of phosphate-buffered saline (pH 7.6) (PBS) with *Triton X-100* at 1 % (PBST at 1 %) incubation for 1 day. Then, sarcomeric Z-band structures were stained with a primary anti-α-actinin antibody (A7811, Sigma-Aldrich, US, dilution 1:200) for 1 day at 4 °C. Strips were washed with PBST 0.1 % at room temperature (RT) for 1 day, and the secondary antibody conjugated with an Alexa Fluor 594 (ab150108, Abcam, UK, dilution 1:100) was applied for 3 days at RT. Finally, strips were washed with PBST 0.1 % for 1 day and fixed in 1 ml of paraformaldehyde (PFA) 4 % at RT for 5 min to prevent antibody detachment. Strips were optically cleared by homogenizing the refractive index of the tissue using serial incubations in 2 ml of 20, 47, and 68% (vol/vol) 2,2'–thiodiethanol (166782-500G, Sigma-Aldrich, US) in 0.01 molar-PBS (TDE/PBS) each for 1 h at RT while gently shaking. The clearing protocol is adapted from Costantini et al. (2015); the staining protocol was first optimized on mouse heart tissue (details and results of the staining protocol optimization can be found in Supplementary Material, section *A* and Supplementary Figure 1, respectively) and then applied to human cardiac tissue.

A custom-made TPFM was used for volumetric tissue reconstruction as performed in Olianti et al. (2020). A mode-locked Ti:Sapphire laser (Chameleon, 120 fs pulse width, 90 MHz repetition rate, Coherent, CA) operating at 780 nm was coupled with a custom-made scanning system based on a pair of galvanometric mirrors (LSKGG4/M, Thorlabs, USA). The laser was focused onto the specimen by a refractive index tunable 25X objective lens (LD LCI Plan-Apochromat 25X/0.8 Imm Corr DIC M27, Zeiss, Germany), having numerical aperture (na) of 0.8 and free working distance (FWD) of 0.57 mm. The system was equipped with a closed-loop *XY* stage for the radial displacement of the sample and with a closed-loop piezoelectric stage for the displacement of the objective along the *Z* axis. Emission filter of 618 ± 25 nm was used for Alexa Fluor 594 detection. The fluorescent signal was collected by a GaAsP photomultiplier module and stored as an 8 bit grayscale intensity signal.

The whole volume was acquired by *Z*-stack imaging of adjacent regions of 450 × 450 μm with an overlap of 40 μm, with stack depth equal to the thickness of the strip and with a *Z* step of 2 μm between images. Each frame is composed by 1,024 × 1,024 px, resulting in a pixel size of 0.44 × 0.44 μm. Stacks were merged using a custom software tool for high-resolution volumetric stitching (Mazzamuto, 2021). This software exploits the overlaps between stacks for correcting the μm-scale displacements of the imaging platform along three axes, aligning stacks through local and global optimizations and finally fusing the 3D image in a single TIFF file. A deconvolution process was performed with Huygens Professional version 19.04 (Scientific Volume Imaging, The Netherlands, http://svi.nl), using the CMLE algorithm, with a signal-to-noise ratio (SNR) of 20 and 40 iterations, and the actual Point Spread Function (PSF) of the system estimated by imaging nanospheres with a diameter of 0.1 μm. Details about the PSF measurement can be found in Supplementary Material.

### 2.2. Image Equalization and Segmentation

To uniform and clean the Z-bands signal over different areas and depths of the 3D image, we used the Contrast Limited Adaptive Histogram Equalization (CLAHE) algorithm (Pizer et al., 1987). We tested the algorithm performance with different values of *clip*, ranging from 0.03 (default) to 0.1, and selecting *clip* = 0.08 by trial and error to ensure a strong equalization (Supplementary Figure 4). CLAHE algorithm was applied independently to each stitched frame of the 3D reconstructions.

Contractile tissue was then segmented in the equalized images. Given that the staining in the Z-bands only allows an intensity-based segmentation process that does not capture complex inter- and intra-cellular textures, we used an adaptive quantization of the image pixel intensities in K balanced clusters by means of the K-Means algorithm, achieving a finer segmentation. We selected *K* = 4 by trial and error (Supplementary Figure 6). A dynamic second classification assigned a label to the pixels of each cluster (*Background*, with intensity 0, or *Tissue*, with intensity 255 in a 8-bit image representation) based on the clusters dimensions, in order to correctly manage images with different quantity of tissue (Supplementary Figure 7). The final binary masks were thus smoothed and cleaned with a set of binary morphological operators. Details about the equalization and the segmentation steps are in Supplementary Material. Finally, all equalized frames were cropped with the binary masks obtained by the segmentation process. This results in a 3D reconstruction of the sample contractile tissue, later involved in the fiber orientation analysis.

### 2.3. Analysis of Sarcomere 3D Orientation

**Figure 1:**
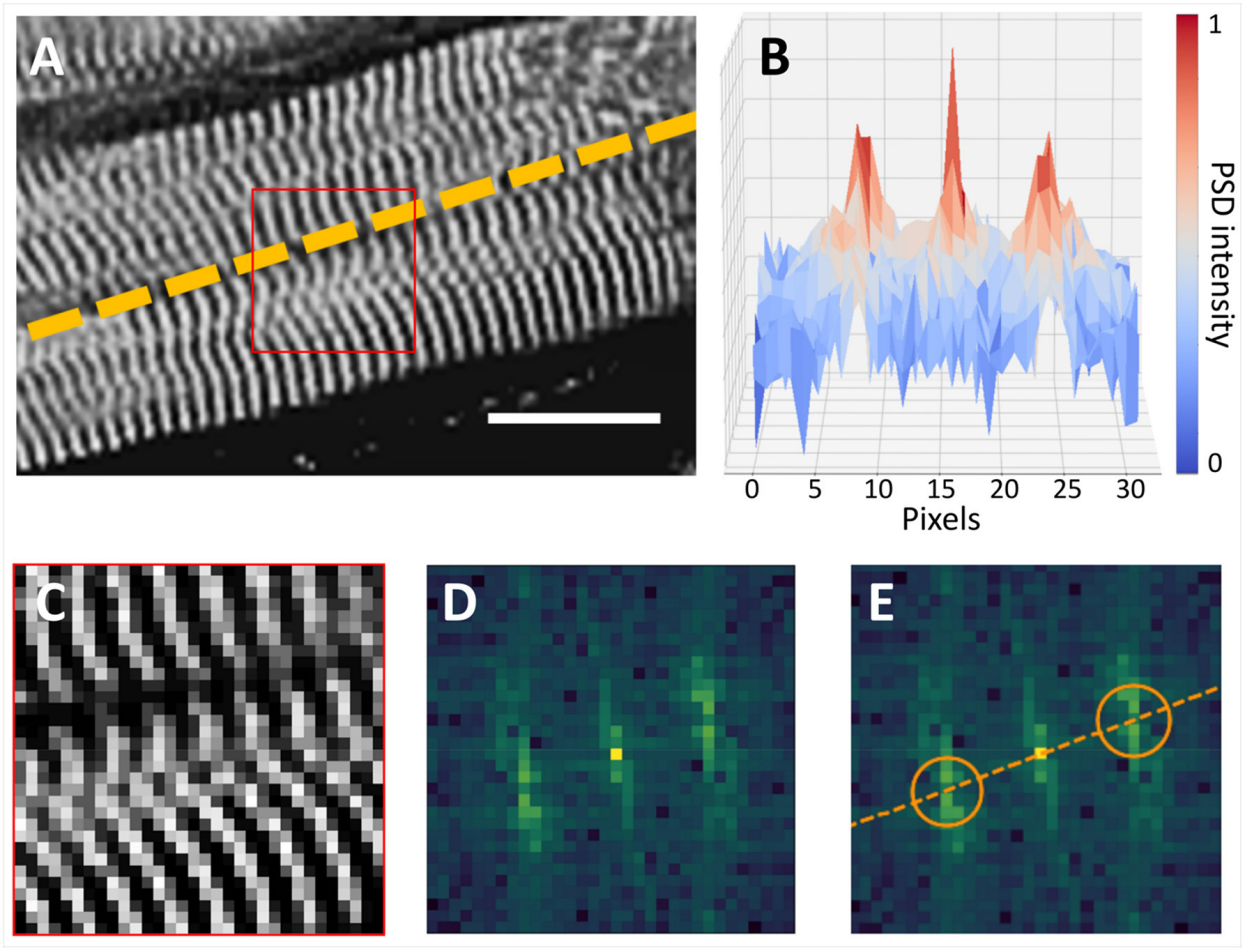
Cardiomyocyte orientation by frequency analysis. **(A)** Cardiomyocyte long axis orientation (dashed line) is perpendicular with Z-bands (Scalebar: 16 μm). **(B)** Intensity profile of the Power Spectrum Density (PSD) of the selection (red square) in **(A)** is shown in the *XZ* plane. **(C–E)** Myocyte orientation in the frequency space: **(C)** the 16 × 16 µm selection of the image in **(A)** is shown with the original pixel size; **(D)** frequency information contained in **(C)**; **(E)** position of frequency peaks is highlighted (orange circles), and original myocyte long axis is restored.

We detected the 3D orientation of a cardiomyocyte portion using the existing perpendicularity between the Z-bands and the cell longitudinal axis, exploiting the periodicity of the sarcomere structures with a frequency analysis approach (Figure 1). Cellular orientation was estimated across the sample with a predefined spatial resolution. In detail, the 3D sample volume delimited by the segmentation mask was virtually dissected in chunks **p** with a size of 16 × 16 × 16 μm. Every chunk was labeled as *tissue* and analyzed only if containing at least the 80 % of contractile tissue to discard borders information.

**Figure 2:**
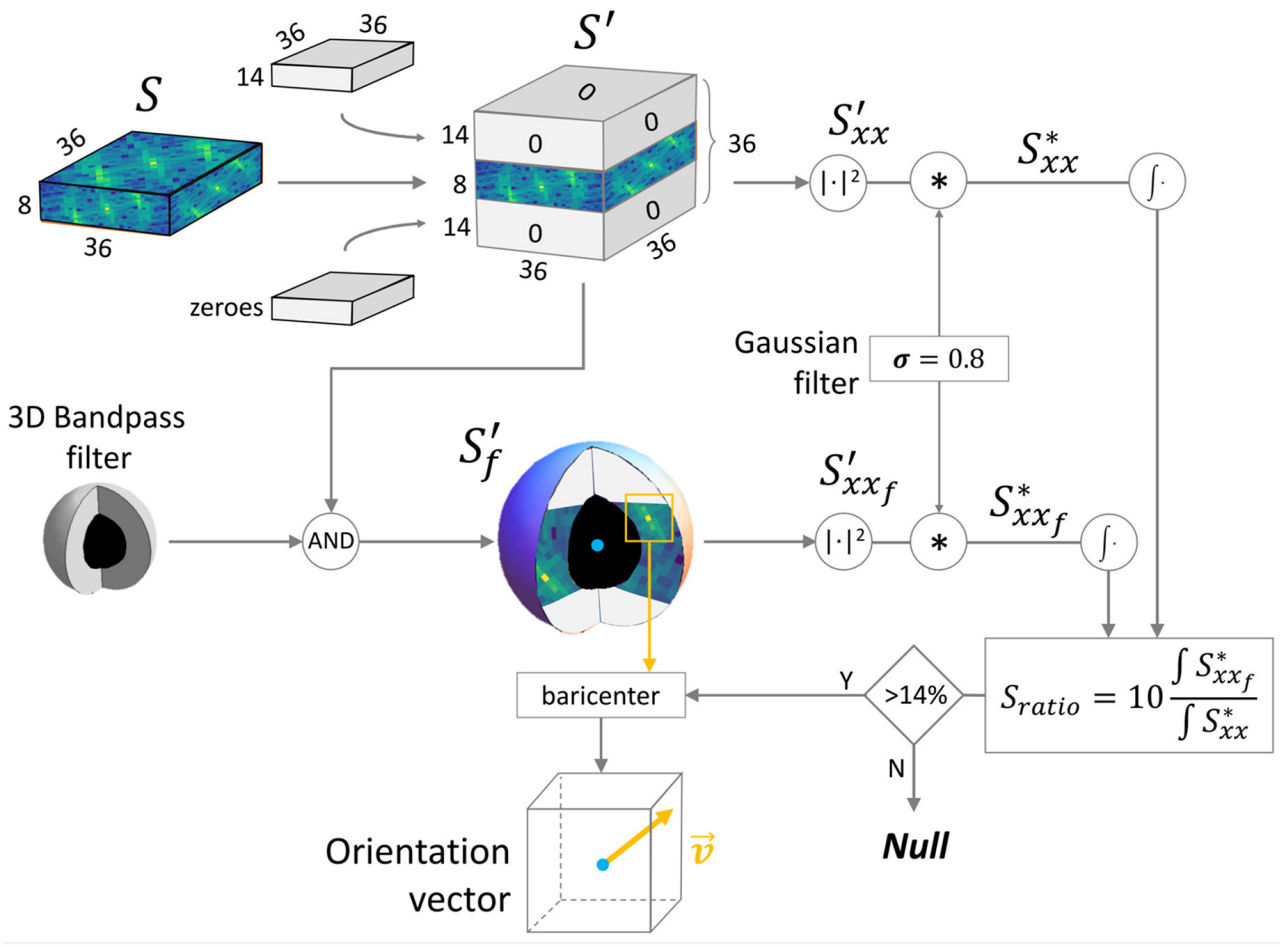
3D spectrum manipulation pipeline. The scheme shows the frequency analysis approach developed to evaluate the reliability of the Z-bands signal and to extract the 3D orientation. S: frequency spectrum of a 16 × 16 × 16 μm virtual sample chunk; $S^{\prime }$: spectrum with isotropic voxel resolution; $S_{f}^{\prime }$: filtered spectrum; $S_{{xx}_{f}}^{\prime }$ and $S_{xx}^{\prime }$: power spectrum densities.

We applied the 3D Fast Fourier Transform (F) to each **p** to extract the spectrum S=F(p) shifted in a symmetric configuration (Figure 2). Both **p** and S have a shape of (36 × 36 × 8) px due the anisotropic resolution. To overcome the anisotropy limit, we used zero-padding in the frequency space, expanding the *Z* axis to obtain a cubic domain. Zero-padding is equivalent (but more efficient) to simulating a voxel size in the spatial domain of 0.44 × 0.44 × 0.44 μm. The padded spectrum $S^{\prime }$ has a shape of (36 × 36 × 36) px, where only the eight central *XY* planes contain the original frequency information. The Z-bands of the human myocytes have a characteristic periodicity with a period *T* typically ranging from 1.6 to 2.0 μm; thus, the bands can be extracted by filtering the spectrum $S^{\prime }$ with a 3D bandpass filter having a cutoff frequency of (1/1.8 μm) = 0.556 μm^−1^ and a bandwidth between 1/(2 μm) = 0.5 μm^−1^ and 1/(1.6 μm) = 0.625 μm^−1^. Due to the selected dimension of the window of 36 × 36 px in the spatial domain, the resolution in the frequency domain was 1/*T*_max_ = 1/(36·0.44 μm) = 0.0631 μm^−1^. Spatial and frequency domain details can be found in Supplementary Material. The filter was implemented in the frequency domain, assigning unitary value at all pixels included in a spherical shell with average radius ρ= 0.556 μm^−1^/0.0631 μm^−1^ ≈9 px and with a thickness between ρ_*m*_= 0.5 μm^−1^/0.0631 μm^−1^ ≈8 px and ρ_*M*_= 0.625 μm^−1^/0.0631 μm^−1^ ≈10 px. All other values are equal to zero. The filtered spectrum $S_{f}^{\prime }$ was calculated by multiplying the binary 3D filter with the expanded spectrum $S^{\prime }$.

The Power Spectrum Density (PSD, defined as $S_{xx}=|S{|}^{2}$) of both $S^{\prime }$ and $S_{f}^{\prime }$ was evaluated ($S_{xx}^{\prime }$ and $S_{{xx}_{f}}^{\prime }$, respectively). To reduce the border contribute in the chunk, *Convolution Theorem* was exploited to efficiently apply a radial smoothing on **p**: A 3D gaussian kernel of (3 × 3 × 3) pixels with σ = 0.8 was convolved with both $S_{xx}^{\prime }$ and $S_{{xx}_{f}}^{\prime }$, obtaining, respectively, $S_{xx}^{*}$ and $S_{{xx}_{f}}^{*}$.

To improve the accuracy of the fiber architecture reconstruction, a test was performed on the frequency domain. We defined:


(1) $$\begin{array}{l} S_{\text{ratio}}:=10\frac{\int S_{xx}^{*}d\omega }{\int S_{{xx}_{f}}^{*}d\omega } \end{array}$$

as a *Signal to Noise Ratio*, i.e., the energy ratio between the frequency contribution of the Z-bands and the total spectrum (ω is the normalized frequency). $S_{\text{ratio}}$ values collected along the sample were normalized between 0 and 1. High $S_{\text{ratio}}$ means high spectral density around the sarcomeres frequencies, i.e., a clear and well-resolved Z-bands periodicity inside **p**. Low $S_{\text{ratio}}$ was associated with a noisy **p**, with not very clear periodicity. Z-bands information was defined *reliable* only if $S_{\text{ratio}}$ is higher than a threshold of 14 % evaluated by trial and error; otherwise, **p** was discarded during the reconstruction of the fiber architecture.

To extract the 3D orientation of the Z-bands periodicity, the principal frequency component (one of the two symmetrical peaks) was localized inside the 3D-filtered PSD $S_{{xx}_{f}}^{*}$ by the discrete position of the maximum intensity voxel **v**_max_. To improve the angular resolution, the real coordinates of the intensity centroid **c** of a (4 × 4 × 4) voxel region around **v**_max_ were evaluated. Finally, the 3D orientation vector $\vec{\text{v}}$ was defined between the center of **p** and **c**. The algorithm exploited the symmetry of the 3D spectrum to represent all the orientation vectors in a congruent way, automatically flipping any vector $\vec{\text{v}}$ with $\vec{v_{y}}<0$ in the *Y*≥0 semi-sphere. A scheme of the reference system can be found in Supplementary Material. The vector length defines the exact frequency of the Z-bands periodicity (i.e., sarcomere length), and its direction defines the cell orientation inside the sample. A final outlier removal step was developed in order to clean the results, comparing each orientation vector $\vec{\text{v}}$ with its neighbors and increasing accordingly the threshold on $S_{\text{ratio}}$. This allowed us to discard outlier vectors with a weak frequency component.

### 2.4. Cytoarchitectonic Reconstruction and Disarray Quantification

The orientation distribution of the cardiomyocytes in the 3D space forms a tensor, named *vector space*, where each position of **p** is associated with its orientation vector $\vec{\text{v}}$. The vector space reassembles the fiber architecture of the whole sample. This reconstruction was analyzed in order to estimate the degree of local tissue disarray, dissecting the vector map in macrovoxels **g**, each including *m* × *m* × *m* orientation vectors.

**Figure 3:**
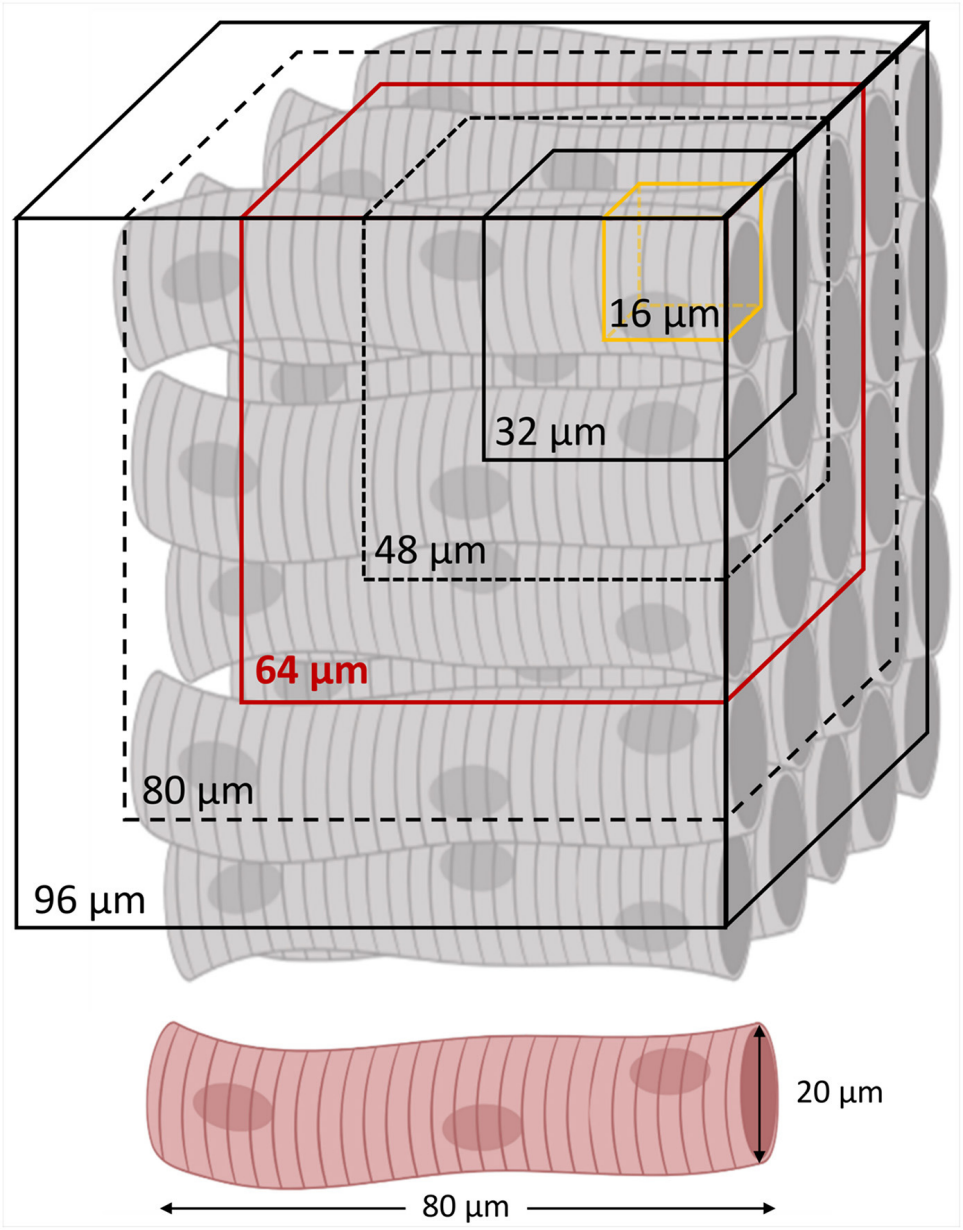
Spatial resolutions of local disarray quantification. A regular cardiomyocyte architecture is schematized with the different spatial resolutions involved in the local disarray quantification. The minimum (a 32 μm side cube) and the maximum (a 96 μm side cube) macrovoxel include, respectively, 2 × 2 × 2 and 6 × 6 × 6 orientation vectors evaluated in a sub-volume of 16 μm sides (in yellow). In red is highlighted the average spatial resolution of 64 μm, selected to create the 3D maps of the local disarray, and for the myocyte angular dispersion estimation by virtual sample comparison.

In order to investigate the dependency between the tissue disorder and the spatial scale of analysis, we performed the local disarray quantification at five different spatial resolutions. In detail, by choosing *m* = 2, 3, 4, 5, and 6, we selected macrovoxels of 32, 48, 64, 80, and 96 μm of side, i.e., ranging from the smallest resolution available (*m* = 2), to a macrovoxel including at least 5 × 5 entire myocytes, each one long about 80 μm (Figure 3).

A macrovoxel is labeled as *valid* if it contains at least 50 % of *reliable* orientation vectors $\vec{\text{v}}$. The total number of valid macrovoxel in the reconstruction is defined as *N*_*v*_. For each valid g∈G, the local disarray and the local alignment along the traction axis of the strip were evaluated.

#### 2.4.1. Local Disarray

*Local disarray* quantifies the misalignment degree of nearby orientation vectors with respect to the mean direction. For each macrovoxel **g**, we estimated the mean direction ${\bar{v}}_{g}$ by normalizing and averaging the *n*_*g*_ reliable elements $\vec{v_{i}}\in \text{g}$. In case of purely random orientations, we expected a *zero* value of $\left|{\bar{v}}_{g}\right|$, while a perfect arrangement of all orientation vectors provides a unitary value. We defined the *local disarray* **d**_*g*_ of each macrovoxel **g** as:


(2) $$\begin{array}{l} {\text{d}}_{g}=\left(1-|{\bar{v}}_{g}|\right)\%. \end{array}$$

Evaluating **d**_*g*_ values across the whole strip, the automatic pipeline creates a 3D disarray map. A gaussian kernel with σ = 10 μm was applied on the map to smooth the 3D visualization.

#### 2.4.2. Local Alignment

Local alignment quantifies the alignment degree of the fibers along the longitudinal axis of the tissue strip (*Y*). For each g∈G, we defined the *local Alignment* **a**_*g*_ as:


(3) $$\begin{array}{l} {\text{a}}_{g}=\frac{1}{n_{g}}\overset{n_{g}-1}{\underset{i=0}{\sum }}\vec{v_{y}}. \end{array}$$

i.e., the average *y* component of the reliable vectors in **g**.

#### 2.4.3. Global Disarray and Alignment

Global statistics of the sample as *Global Disarray* (D) and *Global Alignment* (A) was computed by simply averaging the *N*_*v*_ *local disarray* and *local alignment* values, respectively:


(4) $$\begin{array}{l} D=\frac{1}{N_{\text{v}}}\overset{N_{\text{v}}-1}{\underset{g=0}{\sum }}d_{g}\;;\;A=\frac{1}{N_{\text{v}}}\overset{N_{\text{v}}-1}{\underset{g=0}{\sum }}a_{g}. \end{array}$$

#### 2.4.4. Statistical Analysis

To compare disarray distributions at different spatial resolutions and in different samples, we evaluated the Probability Density Function (PDF) of the **d** values. Independently from the spatial resolution used, local disarray was a set of random, positive, and real values. Log-normality of **d** values distributions was verified with the D'Agostino-Pearson test (D'Agostino and Pearson, 1973).

We used the log-normal PDF defined as:


(5) $$\begin{array}{l} PDF=\frac{1}{x\sigma \sqrt{2\pi }}\exp\left(-\frac{{\left(\ln x-\mu \right)}^{2}}{2\sigma^{2}}\right) \end{array}$$

to fit the distribution. Finally, we calculated *mode* and *standard deviation (std.dev)* of the PDF with:


(6) $$\begin{array}{l} \{\begin{array}{l} mode=\exp\left(\mu -\sigma^{2}\right)\\ std.dev=\sqrt{\left[\exp\left(\sigma^{2}\right)-1\right]\exp\left(2\mu +\sigma^{2}\right)} \end{array}. \end{array}$$

### 2.5. Virtual Sample Generation

**Figure 4:**
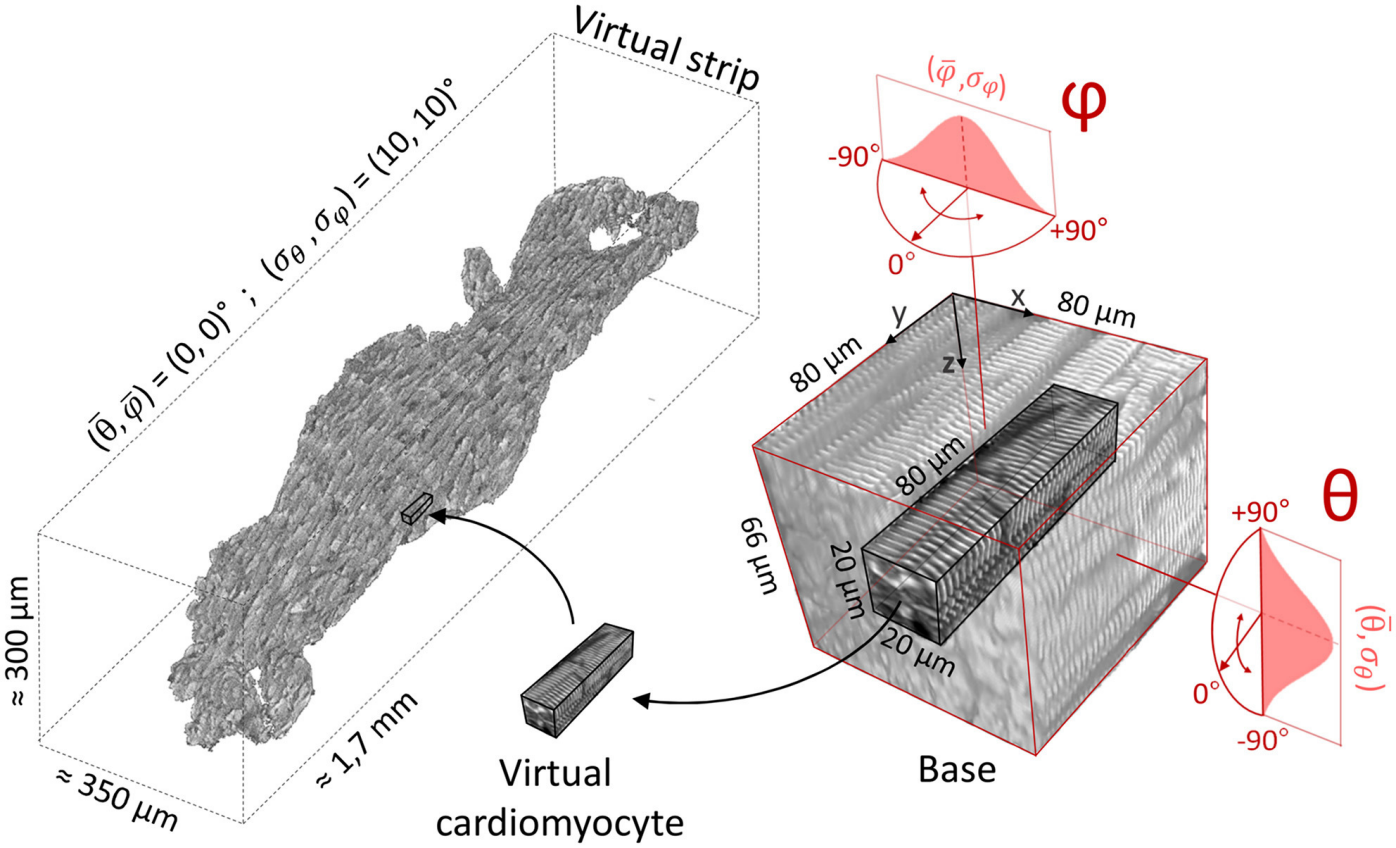
Virtual sample generation. On the right, a representative 3D portion (base) of a Z-bands reconstruction previously aligned with Y axis. Virtual cardiomyocytes are extracted from the base and inserted in the silico sample (on the left). Base rotations are randomly selected from two normal distributions (here, $\bar{\theta }=\bar{\varphi }=0^{\circ }$ and $\sigma_{\theta }=\sigma_{\varphi }=10^{\circ }$).

The virtual sample generator is a simulation tool that generates virtual samples of striated muscle tissue (Figure 4); this is useful in the estimation of the tissue actual geometry of human samples by linking real angular dispersion of myocytes with the theoretical local disarray parameter. The silico tissue was composed by virtual cardiomyocytes, defined as volumes of 80 × 20 × 20 μm of Z-bands signal. For each virtual cardiomyocyte, a 3D rotation—defined by (θ, φ)—was applied to a representative 3D portion (base) of 80 μm × 80 μm × 66 μm of a two-photon muscle sample reconstruction, previously aligned with the Y axis. A blurring operation was applied to the base along the Z-axis after the 3D rotation to simulate the resolution of the optical system along the Z-axis in the rotated *base*. In detail, we used a 2D Gaussian kernel with $\sigma_{k}=\sqrt{\sigma_{z}^{2}-\sigma_{xy}^{2}}$, where σ_*z*_ and σ_*xy*_ are the standard deviation (SD) of PSFz and PSFxy, respectively. The virtual cardiomyocyte was then extracted from the base and inserted in the silico sample. To simulate a random disorganization of the virtual cytoarchitecture, rotations were randomly selected from two normal distributions $\left(\bar{\theta },\sigma_{\theta }\right)$ and $\left(\bar{\varphi },\sigma_{\varphi }\right)$, allowing us to modify the main alignment and the local disarray of the virtual tissue (σ = 0 means uniform cell alignment, i.e., no disarray). This mechanism allowed us to free tune the tissue properties of the virtual samples: $\left(\bar{\theta },\bar{\varphi }\right)$ referred to the orientation of the main force production axis of the muscle, while (σ_θ_, σ_φ_) referred to the misalignment degree of the cellular architecture. Finally, the borders of the cardiomyocytes composing the virtual sample were smoothed with a Gaussian kernel with σ = 1 pixel to clean discontinuities between nearby virtual cells.

## 3. Results

We applied the proposed experimental pipeline on five human heart tissue strips: three dissected from the interventricular septum of a healthy donor as controls (IVS1, IVS2, and IVS3) and two from the atrium of a patient with chronic atrial fibrillation as pathological (AF1 and AF2).

### 3.1. Clearing, Staining, and Imaging

**Figure 5:**
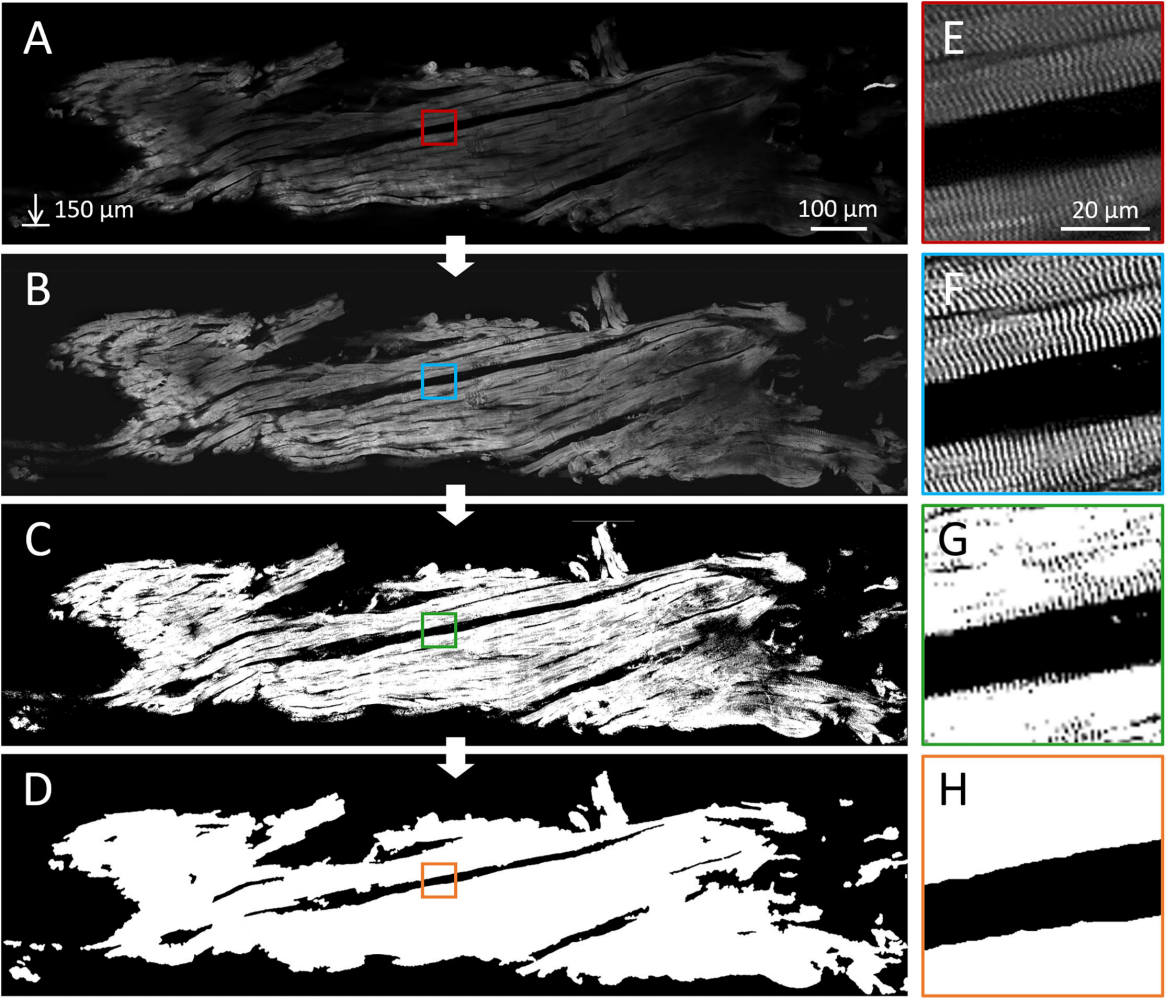
Imaging and segmentation results. Reconstruction and segmentation of the contractile fibers in a human septal tissue strip, labeled with an anti-α-actinin antibody and imaged with a two-photon fluorescence microscope. **(A)** A representative sagittal plane of the reconstruction (Scale bar: 100 μm, depth: 150 μm, pixel size: 0.44 μm). **(E)** Sarcomere Z-bands are visible in the whole strip at sub-micrometer resolution. Results of the main steps of the image processing pipeline are shown: deconvolution and equalization **(B,F)**, binarization by means of the pixel classification **(C,G)**, and the final tissue segmentation **(D,H)** obtained by smoothing the previous result.

We successfully optimized the clearing and α-actinin staining protocol, first on massive samples of mouse heart tissue (Supplementary Figure 1), and later on the human tissue strips to highlight sarcomeric Z-bands. A custom-made TPFM was used to entirely reconstruct each sample with a voxel size of 0.44 × 0.44 × 2 μm and a resolution of 3.1 μm in the Z-axis in about 1 h. A representative frame of a reconstruction is shown in Figure 5A, demonstrating how the optimized clearing and staining protocol allowed Z-bands to be resolved across the whole strip.

### 3.2. Image Pre-Processing and Segmentation

Correct segmentation of the myocardial tissue and the identification of the 3D fiber orientation across the whole strip required a uniform and clear signal of Z-bands over the volume. An image pre-processing pipeline (combining the 3D deconvolution step and the 2D CLAHE-based equalization algorithm) was applied to the TPFM reconstructions (Figure 5). First, deconvolution improved the contrast of the sarcomeric structures; second, equalization corrected the image brightness heterogeneity (Figure 5B). The sarcomeric Z-bands thus resulted well enhanced and resolved across the whole tissue (Figure 5F).

Later, the software pipeline performed the two-step segmentation process selecting the contractile tissue inside each frame (Figures 5C,G) and smoothing the result to correctly label intra- and extra-cellular spaces as *tissue* and *background* respectively (Figures 5D,H). Change the *clip* value during the equalization does not affect the segmentation results (Supplementary Figure 12). Creating a 3D tissue mask by stacking the binary frames, the pipeline was able to isolate the contractile tissue by the 3D two-photon sample reconstruction, allowing us to directly apply the cytoarchitecture analysis.

### 3.3. Fast Fourier Transform-Based Orientation Analysis and Local Disarray Assessment

One of the main challenges of this study was the development of an automatic and reliable approach to extract the 3D orientation of cardiomyocyte portions along the entire volume using fluorescent Z-band signal. We opted for an FFT-based approach exploiting sarcomere periodicity. We selected a cubic tissue chunk with dimension of 16 × 16 × 16 μm as elementary unit to estimate the local cellular long-axis orientation.

**Figure 6:**
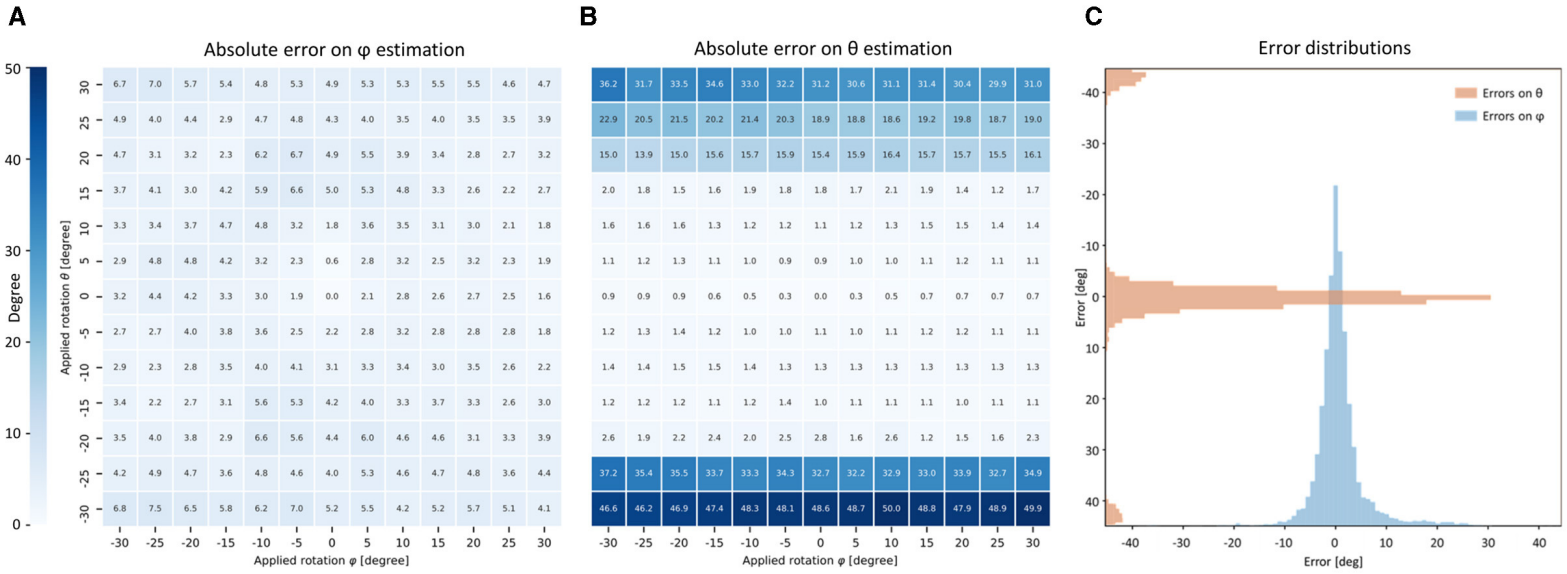
Accuracy of FFT-based orientation analysis. **(A,B)** Tables show the performance of the FFT-based orientation analysis averaged on 140 portions of 16 × 16 × 16 μm randomly extracted from three IVS strips. Virtual rotations on *XY*-plane (*azimuth* φ) and out-of-plane (*elevation* θ) are applied to each portion of Z-bands signal. The absolute error of the proposed method is shown in degree for both φ and θ. **(C)** Relative errors are grouped for all the 13 × 13 virtual rotations applied on the 140 portions and shown as distributions.

We evaluated the performance of the orientation analysis by applying the FFT-based pipeline on a set of tissue portions. In detail, a total of 140 tissue chunks were extracted randomly from the three control strips. A set of 3D rotations—each one defined by polar coordinates (θ, φ)—were applied to each chunk. The 3D orientation was then extracted and represented in the same reference system as (θ′, φ′). Errors between the applied rotation (θ, φ) and the estimated orientation (θ′, φ′) were evaluated for both polar coordinates. Absolute errors averaged over the 140 portions are shown in Figures 6A,B for each virtual rotation (θ, φ) tested. We found that the error associated with the *azimuth* angle φ was uniformly distributed with an average of (3.9 ± 1.3)°. On the other hand, the errors associated with the *elevation* angle θ showed a significant discontinuity where a rotation over 20° is applied. This behavior depends on the resolution limit of the imaging system. The FWHM of the PSF is 3.1 μm in the *Z* axis, while Z-bands structures in our samples have an average period of about *T*= 1.8 μm. Defining $D=\frac{T}{\sin\theta }$ the projection of two consecutive Z-bands on the optical axis *Z*, the system can resolve sarcomere structures with *D* > 3.1 μm. Moreover, by the *Nyquist-Shannon* theorem, is needed *D* > (2.3· 2 μm) to correctly sampling the periodical signal of Z-bands. This intrinsically defined a theoretical upper limit of θ = 23° beyond which Z-bands cannot be resolved (Supplementary Figures 10, 11). Based on these considerations, by restricting the rotation in the range θ ∈ [−20°, +20°], we found an average error on θ′ of (2.9 ± 4.5)°, confirming the good accuracy of the proposed method when Z-bands are optical resolved.

To exclude a systematic error in the orientation analysis, the performance obtained over all the chunks was accumulated and grouped for all the applied rotations (θ, φ) (see Figure 6C). Both distributions were centered at zero, excluding a bias in the orientation analysis process. The θ error distribution showed two little spikes with absolute errors higher than 40°. This behavior may be explained as an aliasing effect caused by the frequency resolution limit in the Z-axis previously described.

**Figure 7:**
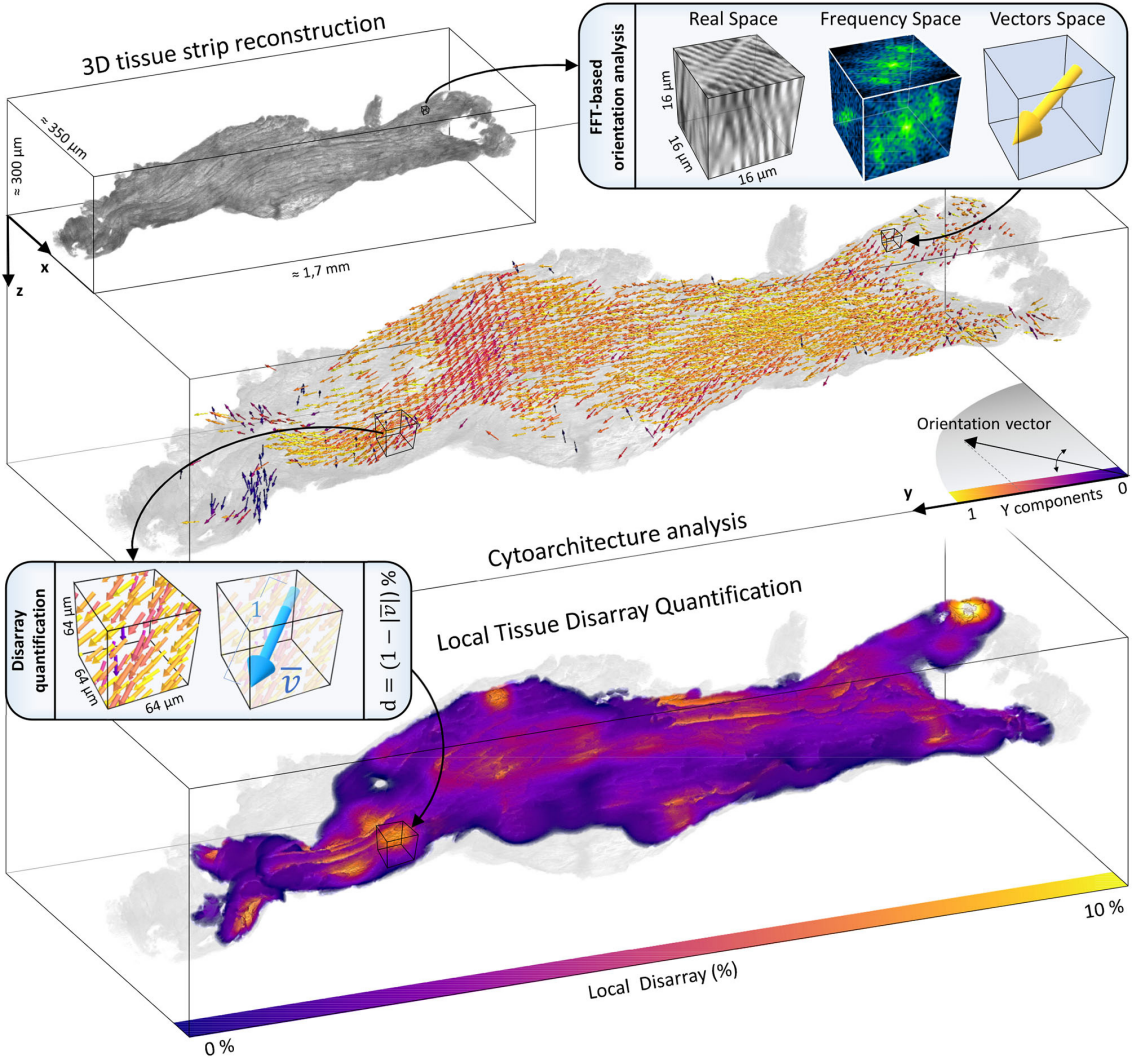
Cytoarchitecture reconstruction and disarray mapping. The 3D reconstruction of a representative strip (IVS1) is shown. The volume is virtually dissected in chunks of 16 × 16 × 16 μm. The FFT-based analysis is applied to each portion to extract the local fiber orientation. The entire cytoarchitecture is collected and represented in a vector space. Vectors color represents the alignment degree with the main axis of force production (*Y*). Then, local cellular disarray **d** is evaluated with a pre-defined resolution (here of 64 × 64 × 64 μm), and a 3D map of the cell misalignment is created smoothing the results with a gaussian kernel with σ = 10 μm.

To assess the tissue organization of the strip, the pipeline applied the orientation analysis to the entire TPFM reconstruction previously segmented and stored the result as a vector map (Figure 7). This 3D vector map was used by the pipeline to evaluate the local vectors misalignment along the entire sample. To this aim, we evaluated a *local disarray* parameter **d**, defined by Equation (2), at a predefined spatial resolution, resulting in a 3D map of the tissue disarray.

### 3.4. Characterization of Local Disarray in Human Cardiac Samples

**Figure 8:**
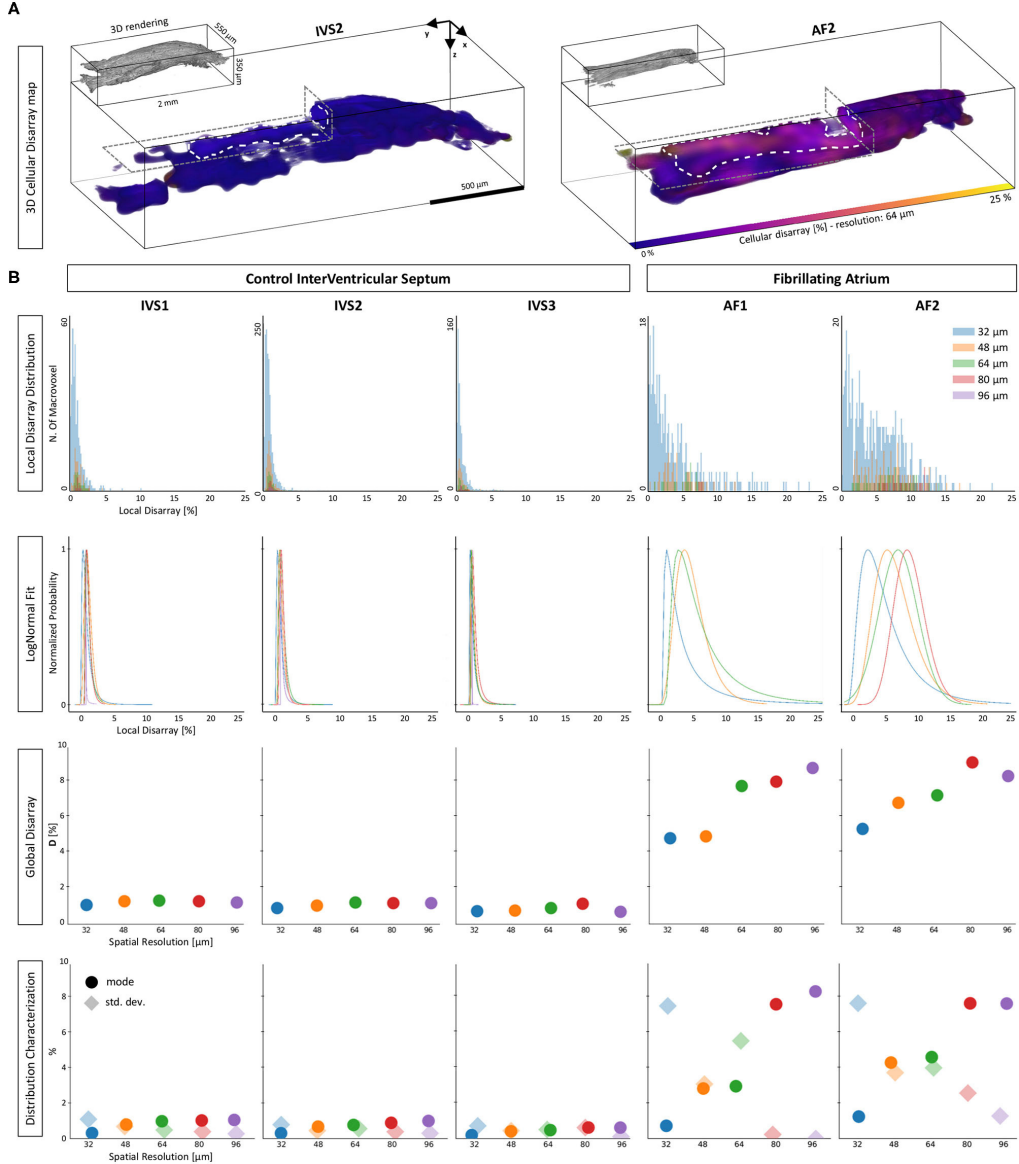
Disarray characterization in control and pathological samples. **(A)** Two representative tissue disarray maps of a control (IVS2) and a pathological (AF2) cardiac tissue strip are evaluated with a spatial resolution of 32 μm. A virtual cut (gray dashed lines) is applied to both maps to show internal sections (white dashed lines). Maps show clear differences between healthy and pathological tissue. **(B)** Results of a multi-resolution cellular disarray investigation applied on three septum tissue strips (IVS1, IVS2, and IVS3) and two fibrillating atrium tissue strips (AF1 and AF2) strips are shown. The raw distributions of the local disarray values are shown. A log-normal PDF is used to fit disarray distributions, and results are normalized separately for each spatial resolution. Global statistics are shown as Global Disarray (D), mode, and SD of the distributions.

The proposed investigation was applied to the five human tissue strips. As a first step, we evaluated and visualized the local disarray with a spatial resolution of 64 μm. Two representative maps of one control and one pathological strip are shown in Figure 8A. A preliminary observation highlighted a high regularity of the tissue arrangement in the IVS preparation, while we observed higher local disarray values in the AF tissue with an heterogeneous distribution across the volume. A systematic local disarray analysis was then performed by quantifying the disarray at different scales by varying the macrovoxel dimension. As described, we choose five spatial resolutions: 32, 48, 64, 80, and 96 μm (Figure 3), leading to a single macrovoxel of 32,768, 110,592, 262,144, 512,000, and 884,736 μm^3^, respectively. Local disarray distribution (LDD) was generated using these spatial resolutions for each sample, as shown in Figure 8B. LDDs are log-normally distributed (Supplementary Table 1) both in control and pathological samples at every spatial resolution. By fitting and normalizing LDDs, we found that while in control PDFs have near-identical shapes at each spatial resolution, in the pathological tissue we found that PDFs are dependent on the spatial resolution used. These results are in accordance with the high degree of disorder that is expected to be found in pathologically remodeled AF tissue.

Quantitatively, for each strip and each spatial resolution used, we quantified Global Disarray (D) from raw data and the mode and the SD from the local disarray PDFs. The difference between the two classes was evident: D is resolution-independent in control strips (with values between 0 and 2%), while in pathological strips D rises by increasing the spatial resolution, with a range between 4 and 10%. Also, the mode and the SD of the LDD confirmed the same trend: in controls, we found very low values of mode and SD, with no significant variation across different scales. This may indicate a homogeneous and well-aligned organization of the myofilaments across the sample. On the other hand, in pathological strips we found low mode and high SD when the analysis was performed at high resolution (small macrovoxel), and we observed an increment of mode and a reduction in SD by reducing the spatial resolution (big macrovoxel). Although this behavior could be interpreted in terms of the cellular disarray expected in pathological atrial tissue, a quantitative estimation of the actual 3D angular dispersion of myocytes occurring in these tissue strips required additional effort.

### 3.5. Cellular Angular Dispersion Assessment

Our preliminary investigation on pathological strips suggests the presence of cellular misalignment, with different local disarray distributions at different scales of analysis. To estimate the actual 3D angular dispersion of the myocytes, we developed a software tool able to create virtual cardiac tissue with a tunable cell misalignment degree based on real TPFM images. The virtual samples were generated by randomly rotated real cardiomyocytes with a predefined angular distribution.

**Figure 9:**
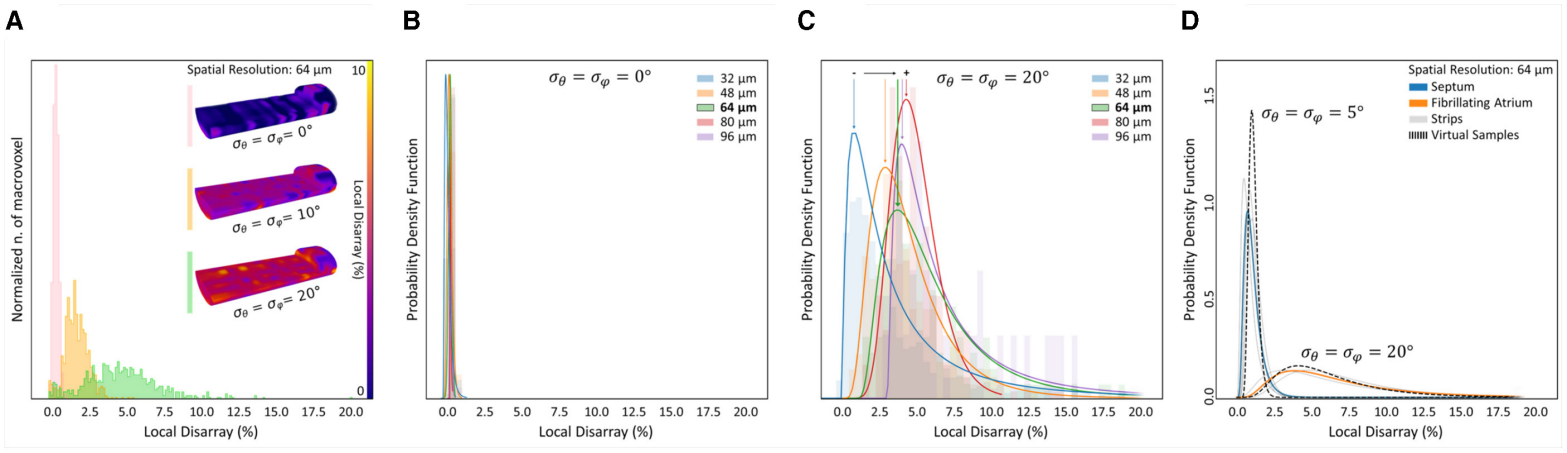
Evaluation of cellular angular dispersion by means of virtual samples generation. Artificial samples of 1,300 × 400 × 280 μm were generated with different (σ_θ_, σ_φ_) values to estimate the actual 3D myocyte angular dispersion by the local disarray distributions found in control and pathological samples. **(A)** Disarray analysis is performed with a fixed resolution of 64 µm on three artificial samples created with $\sigma_{\theta }=\sigma_{\varphi }=0^{\circ }$, 10°, and 20°. For each sample, the 3D map of cellular disarray is shown partially cutted along the image plane, and the distributions of the local disarray values are shown. **(B,C)** The dependence between disarray distribution and spatial resolution is confirmed only in disordered citoarchitectures using log-normal PDF to fit disarray in two virtual samples with $\sigma_{\theta }=\sigma_{\varphi }=0^{\circ }$ (ordered, **B**) and 20° (disordered, **C**). **(D)** Log-normal PDF of cellular disarray at 64 µm is shown for each cardiac tissue strip (gray curves). Average PDF is calculated for control (blue) and pathological (orange) strips. The cellular angular dispersion in the tissue is extracted comparing the disarray PDF of two artificial samples with $\sigma_{\theta }=\sigma_{\varphi }=5^{\circ }$ (control) and 20° (pathological).

First, we generated a set of virtual tissue strips of 1,300 × 400 × 280 μm, with $\bar{\theta }=\bar{\varphi }=0^{\circ }$ (in order to reproduce the preferential cellular orientation aligned with the strip main axis, as in the human strips) and with different degree of cell misalignment (respectively, with $\sigma_{\theta }=\sigma_{\varphi }=0^{\circ }$, 10°, and 20° of myocyte angular dispersion). Second, we applied the cytoarchitecture analysis pipeline, and we quantified the local disarray with a spatial resolution of 64 μm (Figure 9A). The distribution of local disarray follows correctly the expected behavior, with mode and distribution widths that increase accordingly with rising artificial misalignment. We also verified the relation between the LDD and the spatial resolution in two virtual samples reproducing a low ($\sigma_{\theta }=\sigma_{\varphi }=0^{\circ }$) and a high ($\sigma_{\theta }=\sigma_{\varphi }=20^{\circ }$) degree of cellular disarray (Figures 9B,C). In perfect agreement with experimental data, we found that local disarray was log-normally distributed in both samples. Furthermore, aligned tissue provided narrow LDD centered near to zero independently from the spatial resolution, while the tissue with high cellular misalignment showed higher modes that increase by increasing the macrovoxel dimension.

Virtual samples confirmed thus the capability to simulate different cardiac cellular arrangement in a reliable way, producing LDD comparable with the human tissue strips at different tissue regularity degrees and spatial resolutions. Based on this, we employed virtual samples to reproduce the LDD found in control and pathological tissue, in order to assess the actual cellular arrangement. We evaluated the mean local disarray PDF found in IVS and AF strips at the spatial resolution of 64 μm, and we generated a set of virtual samples to establish which cellular angular dispersion produce a virtual PDF that best match real data. We found that IVS and AF tissue classes are characterized by a cellular angular distribution of σ = 5° and σ = 20°, respectively (Figure 9D).

## 4. Discussion and Conclusion

In this study, we employed an optimized clearing and staining protocol, combined with TPFM imaging and an image pre-processing pipeline, to reconstruct millimeter-sized myocardial strips with micrometer spatial resolution. Staining with the α-actinin enabled us to extract the myocyte long-axis orientation with a spatial resolution below the cellular dimension, and to select only tissue with a well-periodic organized Z-bands. This strategy allowed us to reconstruct the effective contractile architecture of the muscle without being affected by any different biological structures as vessels, nerves, or fibrotic structures. An FFT-based orientation analysis was developed to provide a high spatial resolution orientation map with a voxel size of 16 × 16 × 16 μm.

The challenge of reconstructing massive samples with micrometer resolution was tackled with a tissue clarification protocol. However, imaging massive samples requires objectives with sufficient FWD, i.e., relatively low numerical aperture. Our solution allowed us to reconstruct samples with depths up to 500 μm, however limiting the axial resolution of the optical system to approximately 3.1 μm. Z-bands of high-tilt myocytes were not resolved because of this structural limitation. However, this did not prevent a reliable reconstruction of the contractile tissue. Moreover, selecting a voxel depth of 2 micrometers allowed us to maintain an acquisition time of about 1 h for each sample, preventing tissue damage while immersed in the TDE-based refractive index medium, without affecting image quality. In the future, improved axial resolution may lead to reconstruct sarcomeric structures in even more complex cytoarchitecture. Furthermore, the combination of an improved axial resolution, the optimized *alpha*-actinin staining protocol, and the FFT-based image analysis may allow to detect myocyte orientation under the cellular dimension, and provide information about the average length and the regularity degree of the sarcomere structures at micrometric scale, a key factor in the contractile tissue physiology and patho-phisiology.

To test the sensitivity and applicability of the proposed method, the experimental pipeline was applied as proof-of-principle to quantify and characterize the local disarray at different spatial resolutions in two structurally and functionally different classes of cardiac human tissue, where a significant difference in the degree of cell alignment is expected. Healthy tissue of interventricular septum trabecula was used as a control, while the atrium of a patient suffering from chronic atrial fibrillation was used as pathological remodeled tissue.

Preliminary results showed that the local disarray is log-normally distributed in both healthy and pathological tissue. In control, where the cells are expected to be highly ordered, local disarray distribution was not affected by the dimension of the analysis scale. In the pathological strips, the tissue was more disordered, and the disarray distribution depended on the spatial resolution involved. By choosing analysis macrovoxels ranging from a cellular to a multi-cellular spatial scale, we found that, while in control the mean and the mode of the disarray distribution were constant, in remodeled tissue these parameters raised while increasing the spatial scale, reaching a plateau at the highest macrovoxel dimensions. This behavior could be explained by assuming that, in a millimeter-sized tissue portion, the cell-to-cell disarray is the main contributor to the tissue disorganization, confirming the sensitivity of the proposed method.

A comprehensive quantification of tissue disarray is challenging not only for its dependency from the spatial scale of analysis and from the biological structures involved but also due to the itself intrinsic theoretical definition as geometrical parameter. Linking a theoretical parameter of the cytoarchitecture regularity with the actual angular dispersion of myocytes is not trivial in massive samples. In order to provide a direct correlation between the local disarray distribution and the cell-to-cell organization, we proposed a cardiac tissue simulator that employed real single-cell structural data to create virtual tissue strips where the cellular misalignment degree is arbitrarily tunable. In this proof-of-principle, the virtual sample generator was capable to perfectly reproduce the main features of the local disarray distribution found in the human tissue. It matched the experimental result of healthy and pathological tissues by setting the myocyte angular dispersion at 5° and 20°, respectively. In the future, involving a wider range of cardiac tissue samples can confirm the robustness of this new methodological approach, paving the way for more accurate characterizations of the heart contractile muscle.

The presented method overcomes the limits of current techniques, allowing us to estimate the actual myocyte geometrical organization in three dimensions in healthy and diseased massive cardiac tissues. The proposed pipeline allows a quantitative assessment of tissue remodeling and to directly correlate mechanical and energetic measures with the morphological organization of the muscle. Finally, it also enables *in silico* simulation of action potential propagation, by employing a cell-to-cell diffusion model.

## Data Availability Statement

The raw data supporting the conclusions of this article will be made available by the authors, without undue reservation.

## Ethics Statement

The studies involving human participants were reviewed and approved by Careggi University-Hospital (2006/0024713; renewed May 2009). The patients/participants provided their written informed consent to participate in this study.

## Author Contributions

FG, LB, CF, CP, and LS contributed on the conception and design of the study. EL, IC, and GV performed sample preparation and imaging. FG and LB developed the software. FP contributed with the equipment. FG performed image and data analysis. FG and LS wrote the first draft of the manuscript. All authors contributed to manuscript revision, read, and approved the submitted version.

## Funding

This project has received fundings from the European Union's Horizon 2020 research and innovation programme under grant agreement No 952166 (REPAIR), MUR under the FISR program, project FISR2019_00320, and Regione Toscana, Bando Ricerca Salute 2018, PERCARE project.

## Conflict of Interest

The authors declare that the research was conducted in the absence of any commercial or financial relationships that could be construed as a potential conflict of interest.

## Publisher's Note

All claims expressed in this article are solely those of the authors and do not necessarily represent those of their affiliated organizations, or those of the publisher, the editors and the reviewers. Any product that may be evaluated in this article, or claim that may be made by its manufacturer, is not guaranteed or endorsed by the publisher.
