# Supplementary material for: Quantification of Myocyte Disarray in Human Cardiac Tissue
Source: Front Physiol. 2021 Nov 16;12:750364. doi: 10.3389/fphys.2021.750364 (PMC8635020; doi:10.3389/fphys.2021.750364)
Supplement: Supplementary file 1 [file Data_Sheet_1.PDF]

Supplementary materials

A. *Staining protocol optimization*

The staining protocol used on human samples has been previously optimized on strips dissected by three-month-old C57Bl/6 wild-type (WT) mouse heart tissue. The heart was excised, and the proximal aorta was perfused retrogradely with 0.01 M PBS. The heart was fixed and strips were dissected as described in the manuscript for the human strips. Then, we performed trials with primary (Ig1) and secondary (Ig2) antibodies at different incubation times (Fig. S1). We found the staining protocol producing the highest signal amplification correspond to incubation time of 24 and 72 hours for Ig1 and Ig2 respectively.

B. *Software development details*

The software pipeline was developed in Python 3.6.8. All numeric data representation and elaboration were performed using the libraries NumPy 1.14.1 and SciPy 1.4.1 [1], [2]. Equalization step with CLAHE algorithm was performed with the function exposure.equalize_adapthist() from Python library Scikit-image 0.14.0 [3]. The K-Means algorithm employed during the pixel classification was implemented in the library OpenCV-python 3.2.0.8 [4]. Morphological operators applied on the segmentation images were suited in the morphology module of the same library. 3D FFT was performed using the NumPy function np.fft.fftn(). Python graphic libraries used were Matplotlib 3.2.1 (2D), Mayavi 4.7.1 (3D vectors field) and Seaborn 0.9.0 (numerical plots) [5], [6]. 3D rendering of the sample was created with software Fiji [7], [8].

C. *Point Spread Function estimation and Image Deconvolution*

In order to evaluate the Full Width Half Maximum (FWHM) of the actual Point Spread Function (PSF) of the Two-Photon Fluorescent Microscope (TPFM), we simulated materials points smallest than the theoretical FWHM using nanospheres of about 0.1 μm in diameter (INVITROGEN Molecular Probes - FluoSpheres carboxylate-modified microspheres - 0,1 μm, yellow-green fluorescent – [505/515]) dissolved (concentration 1:1000) in a gel composed by 32% of PBS and 68% of TDE to reproduce the refractive index present during the human muscle strip reconstructions. We used the same composition of the solution used with the muscle sample in order to obtain the same refraction index (n = 1.46). The gel preparation (20 ml of solution) is described here: first, we combine 6.4 ml of PBS (32%) with 13.6 ml of TDE (68%) in a 50ml becker. After we added 0.8 g of Agarose gel (Agarose gel OLE, Low Temperature) to gel the solution. In order to melt the Agarose, the solution was heated and continuously mixed for about 5 minutes. When the solution was transparent, we add 20 μl of the microspheres solution (concentration of 1/1000) and posed it in a plate (10 cm in diameter). After few minutes at 4°C, the solution was gelled and ready. One stack of this preparation was acquired with the two-photon microscope with pixel size (0.097, 0.097, 0.5) μm (x, y, z) and with a depth of about 100 μm. Fig. S1 shows a single microsphere in nine image-planes at different z positions. The average shape of the PSF was evaluated using Huygens Software (Huygens Professional, version 19.04, Scientific Volume Imaging, The Netherlands, http://svi.nl). Specifically, the whole stack was loaded into Huygens. First, we set the image parameters (pixel size, scan direction, wavelenght). Later, we used the PSF distiller with default settings to estimate the PSF. Inside our sample of gel, Huygens finds about 30 microspheres and average their shapes. The average PSF was stored to be used during the deconvolution process. The Full Width Half Maximum (FWHM) of the actual PSF reconstructed was (0.690, 0.771, 3.093) μm.

To apply the deconvolution to our two-photon images, we loaded both the data and the PSF in Huygens. Using the Deconvolution Wizard with default settings, we obtained the deconvolved data. Fig. S3 shows a represented deconvolved frame and the distribution of the pixel intensity. Deconvolution decreased brightness in the images.

D. Contrast Equalization

Contrast Limited Adaptive Histogram Equalization (CLAHE) algorithm was applied to each frame of the 3D TPFM reconstruction after the deconvolution process. We selected the CLAHE algorithm because when the percentual increment of intensity was larger than a given threshold (*clip*) CLAHE redistributes this excess energy over all the spectrum. It prevents saturation, increases contrast, and yields a uniform luminosity in the different parts of the image.

The algorithm depends on the value of *clip*, thus we tested the performance with different *clip* values, ranging from 0.03 (default) to 0.1. Higher values of *clip* squeeze more pixels to saturation, while smaller ones do not produce a sufficient increase of the contrast. According with these results (Fig. S3) we selected *clip* = 0.08 to ensure a strong equalization. In Fig. S5 is shown a representative result with the equalized intensity distribution. The dimension of the CLAHE filter kernel was set as 128 px by default. CLAHE algorithm was applied independently to each stitched frame of the reconstruction between the deconvolution step and the segmentation step.

E. Image Segmentation

Image segmentation step was essential to reconstruct the 3D shape of the sample, to limit the fibers orientation analysis to the contractile tissue, and to obtain a high precision estimation of the volume with a micrometer scale accuracy. In the sample, only Z-bands were stained, allowing an intensity-based segmentation process.

However, the Full Width Half Maximum (FWHM) of the PSF of the TPFM used was 0.690 μm × 0.771 μm × 3.093 μm in (X, Y, Z). The non-ideal optical sectioning of about 3.1 μm smoothed the borders of the excited tissue preventing a direct binarization approach. Moreover, a simple threshold segmentation did not consider the complex intra-cellular textures.

To overcome these limitations, we adaptively quantized the pixel intensities $p_j \in [0, \dots, 255]$ in a smaller number of levels by means of the K-Means algorithm. K-Means was used to divide the histogram *H* in K balanced clusters C_1, …, C_k obtaining a flexible quantization of gray levels from [0, …,255] to the cluster's centroids $\{v_i \in [0, \dots, 255] \mid i=1,\dots,K\}$:

$$\begin{cases} \mathbb{H} = \{\, p_j \mid j = 0, \dots N_s \,\} = \bigcup_{i=1}^{k} C_i \\ with \quad \forall i = 1, \dots, K : \\ C_i = \{\, p_j \mid j = 1, \dots, |C_i| \,\} \;\; ; \; \overline{C_i} = v_i \end{cases}$$

where $N_s = X_s \cdot Y_s$ was the number of pixels in each image. After the quantization of the histogram *H*, clusters were ordered by their centroid values:

$$\mathbb{C} = \{C_1, \dots, C_k\} \quad s.t. \quad v_1 < \dots < v_k$$

Finally, we quantized the entire image assigning to each pixel the centroids values of their own cluster:

$$p = v_i = \overline{C_i} \quad \forall p \in C_i \quad \forall i = 1, \dots, K$$

K value was determined by trial and error (Fig. S6).

We have found the best results using K = 4 followed by a dynamic second classification that assign at every cluster a label, *Background* (B) or *Tissue* (T). This second step was dynamically driven by the clusters dimensions to correctly manage images with different quantity of tissue:

$$\begin{cases} B = \{C_1\}, \quad T = \{C_2, C_3, C_4\} \quad if \quad |C_1| > |C_2| \\ B = \{C_1, C_2\}, \quad T = \{C_3, C_4\} \quad if \quad |C_1| < |C_2| \end{cases}$$

In fact, K-Means produces four approximately balanced clusters. In images with a big presence of tissue,

only the first cluster C_1 contains background pixels. By contrary, in images with a small portion of tissue, background pixels were split into two clusters (C_1 and C_2). An example of this mechanism is shown in Fig. S7.

After the dynamic classification, binary masks were created assigning 0 (False) to the background pixels and 1 (True) to the tissue pixels. Because of the high spatial resolution of the original images, often some intra-cellular space was wrongly classified as background. The segmentation result was thus cleaned by a smoothing process applied to each frame. The smoothing steps include the following morphological operators:

- Closing with a disk with radius $r = 2$ px.
- Opening with a disk with $r = 1$ px.
- Removal of salt and pepper noise: first black connected elements (holes) with an area smaller than $T = 0.014$ % of the entire image area were filled (i.e. structures having an area in the order of nm^2); then white connected objects smaller than the same T were deleted.
- Opening with a disk with $r = 3$ px.
- Dilatation with a disk with $r = 1$ px.

Finally, all equalized frames were cropped with the boolean masks obtained by the segmentation process.

Segmentation sensitivity from respect to the CLAHE-based equalization has been evaluated (Fig. S12). The solution appears robust to the equalization parameter variability.

F. *Supplementary figures*

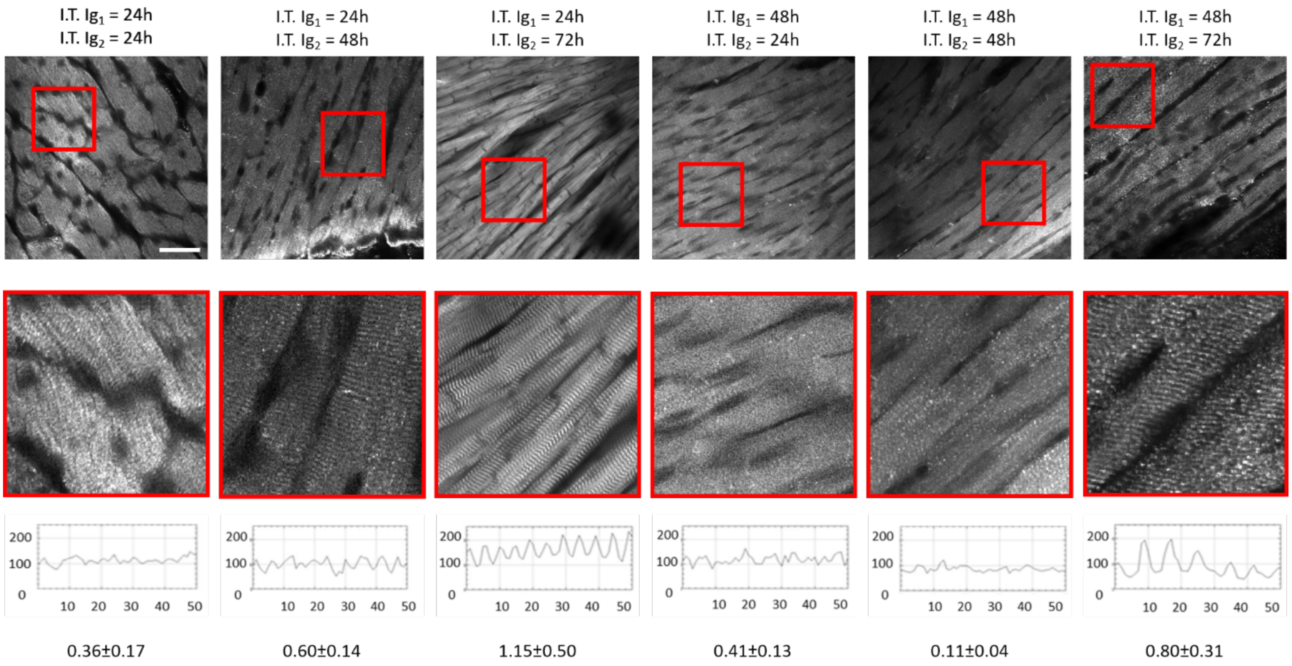

Fig. S1: **Staining optimization.** Representative two-photon images of mouse heart stained with Ig1 and Ig2 antibodies at different incubation times. Red squares are magnifications to appreciate sub-cellular resolution. Plot profile analysis performed with Fiji (http://fiji.sc/Fiji) to assess the best contrast in sarcomeric staining. Best results are obtained with Ig1 and Ig2 of respectively 24 and 72 hours. Scale bar 50 μm.

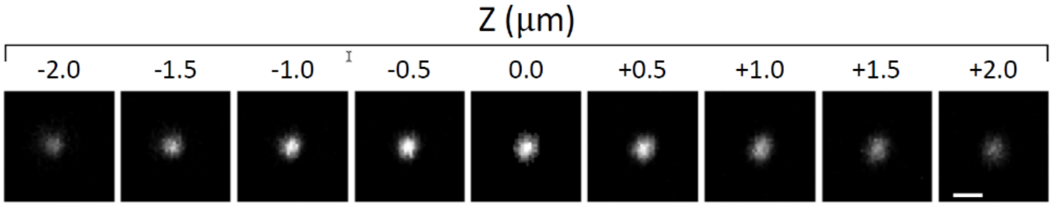

Fig. S2: **Actual Point Spread Function.** Imaging of a nanosphere (INVITROGEN Molecular Probes - FluoSpheres carboxylate-modified microspheres - 0.1 μm, yellow-green fluorescent – [505/515]) imaged with the Two-Photon Fluorescence Microscope (Pixel Size of 0.083 μm × 0.093 μm × 0.5 μm in *XYZ*) shown at nine different depths (Scalebar of 1 μm). The approximation of the Point Spread Function obtained has Full Width Half Maximum of 0.690 μm × 0.771 μm × 3.093 μm in *XYZ.*

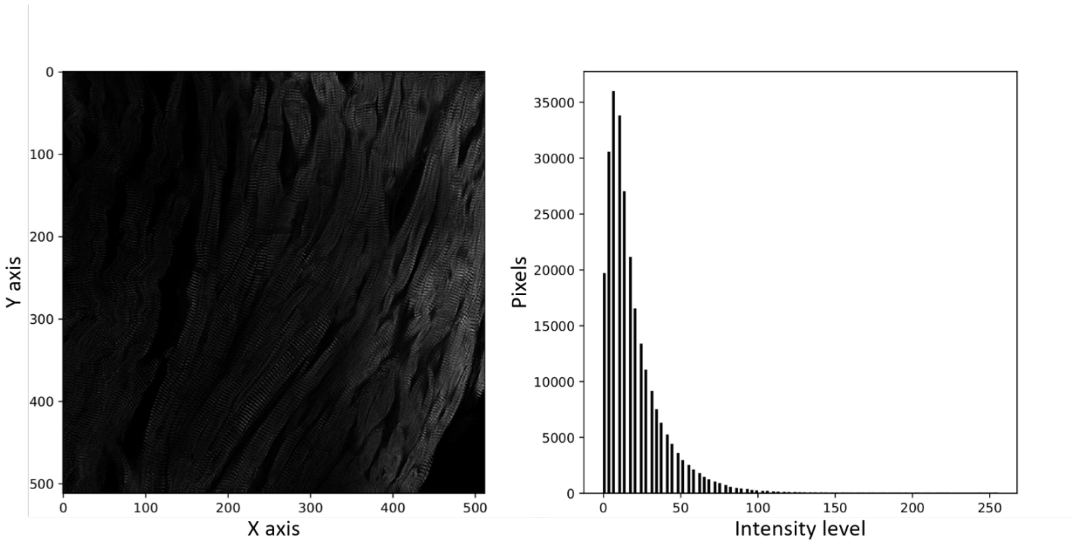

Fig. S3: **Brightness in deconvolved images.** A 225 µm × 225 µm frame of a human cardiac tissue reconstruction after the deconvolution process, with its intensity distribution.

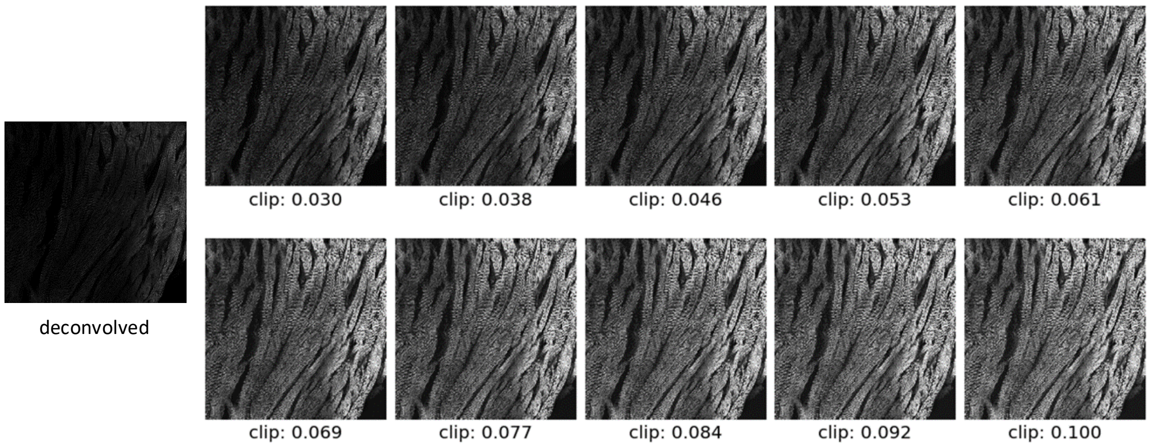

Fig. S4: **CLAHE optimization.** Contrast Limited Adaptive Histogram Equalization (CLAHE) algorithm is applied on a 225 µm × 225 µm portion of a represantive deconvolved frame with different *clip* values.

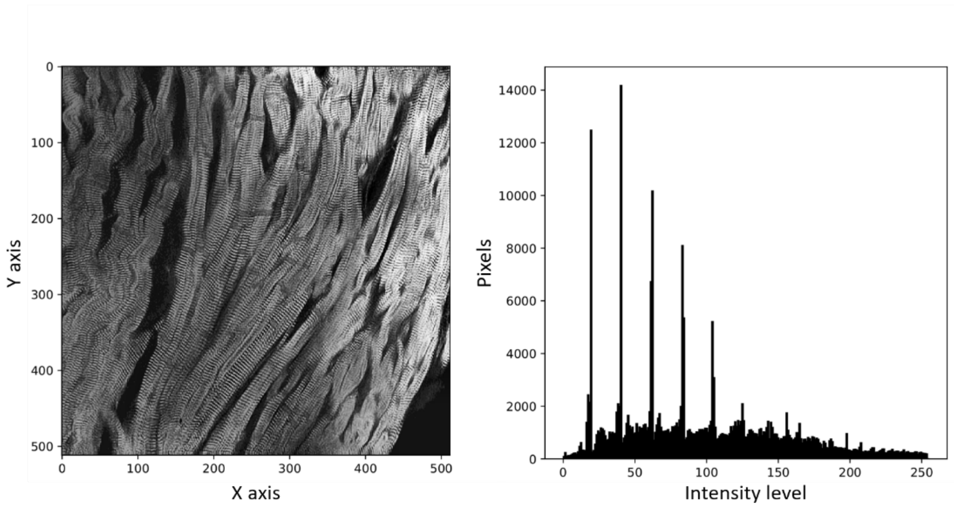

Fig. S5: **Brightness after CLAHE.** The 225 µm × 225 µm portion shown in Fig. S3 is equalized with CLAHE algorithm (clip = 0.8), and the new intensity distribution is shown.

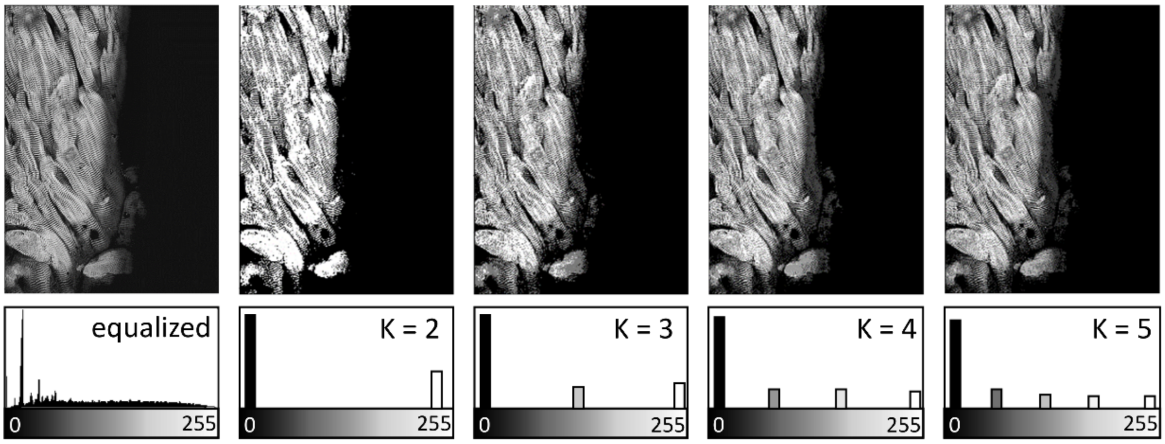

Fig. S6: **Pixel clustering optimization.** On the left is shown a representative 225 µm × 170 µm frame equalized through the CLAHE algorithm with a *clip* value of 0.8 and its histogram. K-Means algorithm is applied to split the pixel intensity values into K = 2, 3, 4, an d 5 groups. Histograms show the resulting clusters. We selected K = 4 to obtain flexibility in the pixel classification, allowing to discard both *background* and *low-intensity* pixels (border of the sample, out-of-focal-plane tissue areas) and collect pixels of contractile tissue.

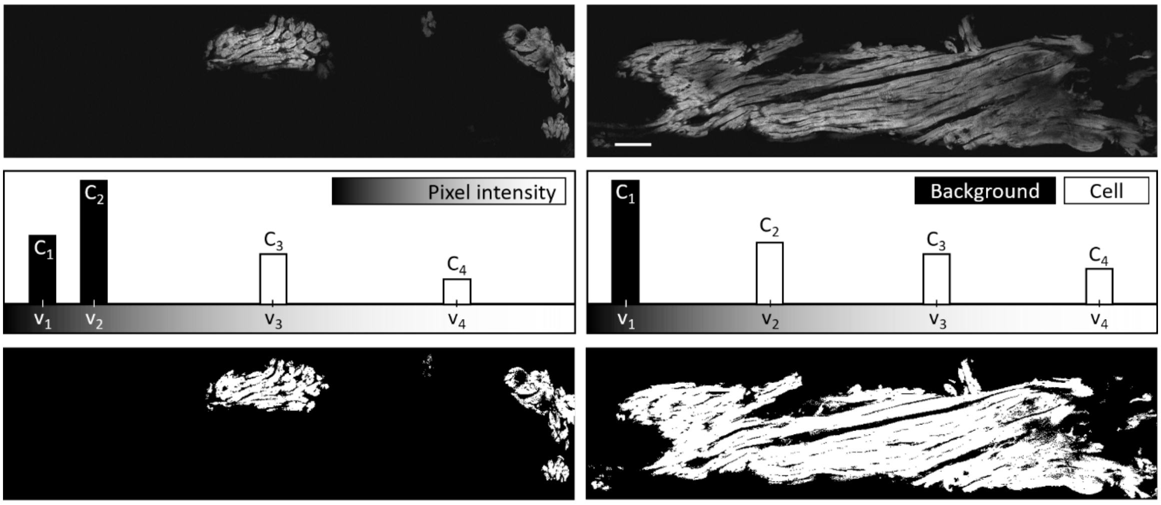

Fig. S7: **Automatic pixel classification.** Above, two representative stitched and equalized frames, containing a small (left) and a big (right) portion of tissue are shown. Below, the pixel intensity quantization is performed with the K-Means algorithm and K=4 clusters. The dynamic and automatic clusters classification labels each cluster as *Background* or *Tissue* accordingly with the ratio tissue/backround present in the frame. At the bottom, the result of the pixel classification. Scale bar: 100 μm.

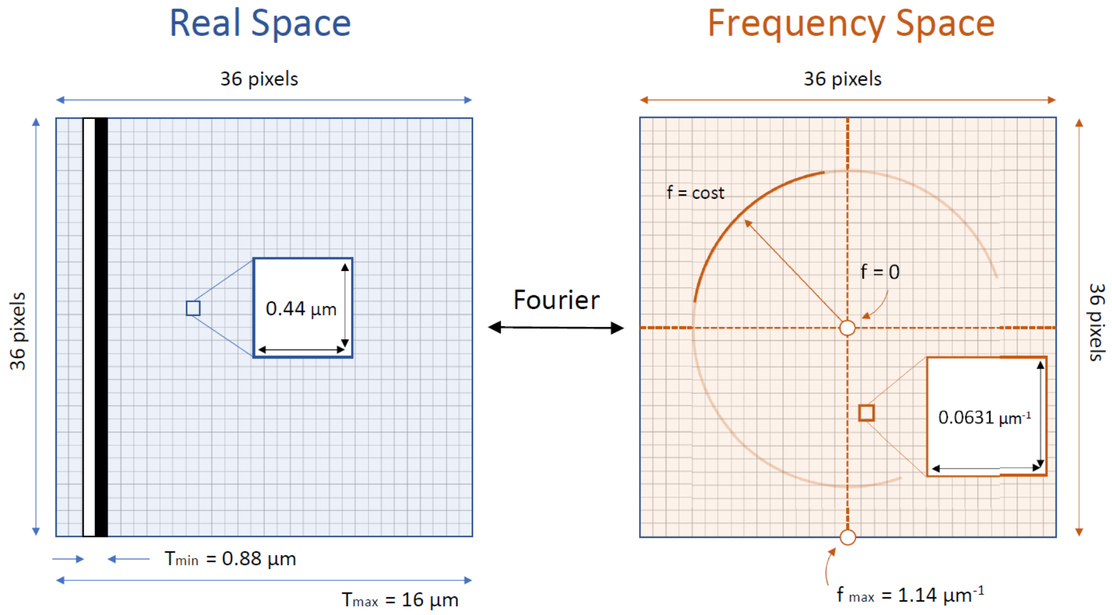

Fig. S8: **Real and frequency space characteristics**. Scheme of a 2D *XY* frame of an elementary chunk (16 μm × 16 μm × 16 μm) represented in both real and frequency domain. In the real space, the Pixel Size (PS) of 0.44 μm allows to sample a periodic signal with period *T* between 0.88 μm (*T* minimum) and about 16 μm (*T* maximum). The two vertical bands (white and black) represent a signal with a period of 0.88 μm. A window of (36 × 36) pixels in the real space allows us to represent frequencies between 0.0631 μm^{-1} (minimum, i.e. PS of frequency domain) and 1.136 μm^{-1} (maximum). Here, all the points on a centered circle have the same frequency.

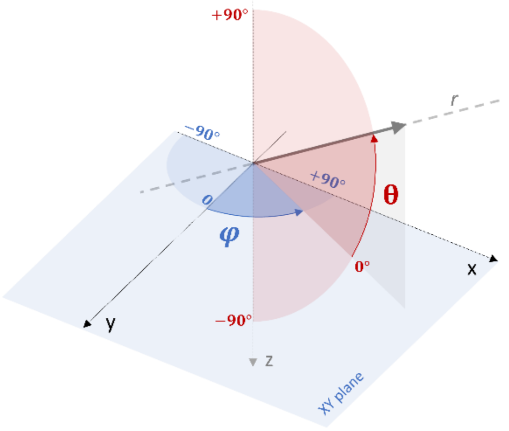

(a) Polar coordinates system.

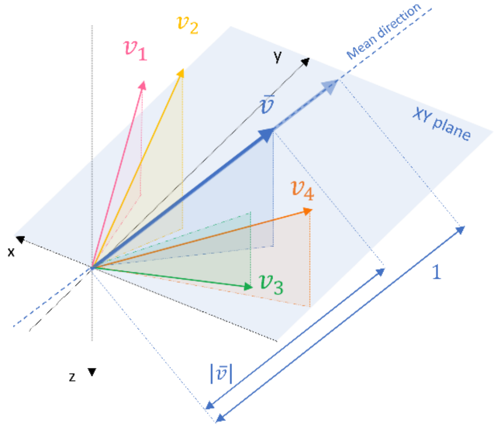

(b) *Local disarray* definition.

Fig. S9: **Reference system and disarray geometrical definition**. In (a) the polar reference system used in this paper is shown (φ: *azimuth*; θ: *elevation*). Domains (φ ∈ [-90°; +90°] and θ ∈ [-90°; +90]) define the semi-space Y ≥ 0 used by the algorithm. In (b) is shown the geometrical representation of *local disarray*. In this example v_1, v_2, v_3 and v_4 are four normalized orientation vectors of four nearby cardiomyocytes. The average vector $\bar{v}$ defines the mean direction of the local fibers. *Local Disarray* is defined as $d = (1 - |\bar{v}|)\%$.

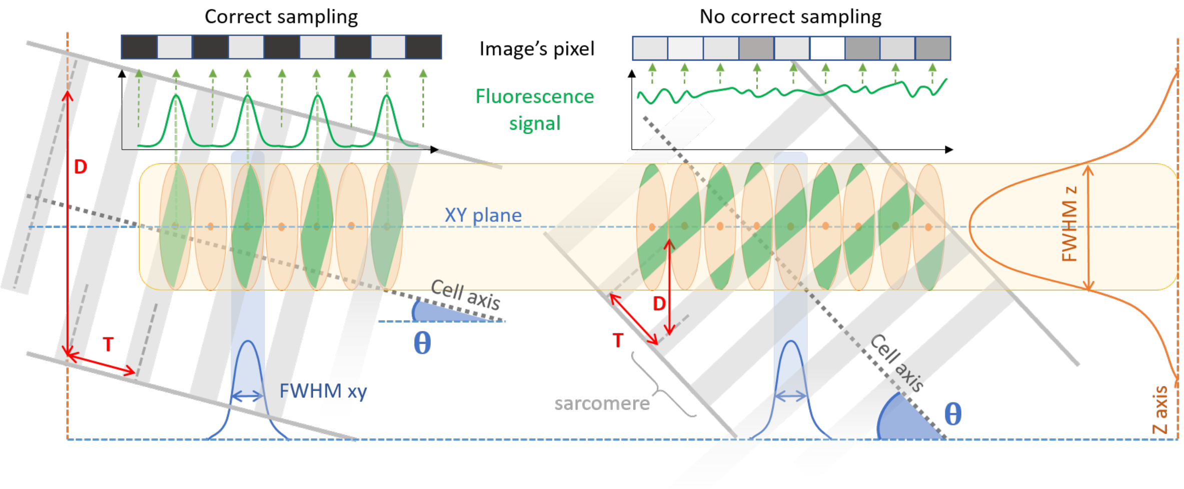

Fig. S10: **Sarcomere sampling of inclined cells**. The scheme represents the excitation and the revelation of cardiomyocyte's Z-bands with a little (on the left) and a big (on the right) angle (θ) between cell long axis and image plane (*XY* plane). Grey bands represent stained Z-bands, locally excited (green portions) by the two-photon microscope with a FWHM of the PSF of 0.771 μm in *XY* (blue gaussian curve) and 3.093 μm in *Z* (orange gaussian curve). With *T* the sarcomere length and *D* the distance between intersections of *Z* axis with two consecutive Z-bands, the sampling limit is satisfied (left) and not (right), i.e. the system is respectively able or not able to resolve and sampling Z-bands.

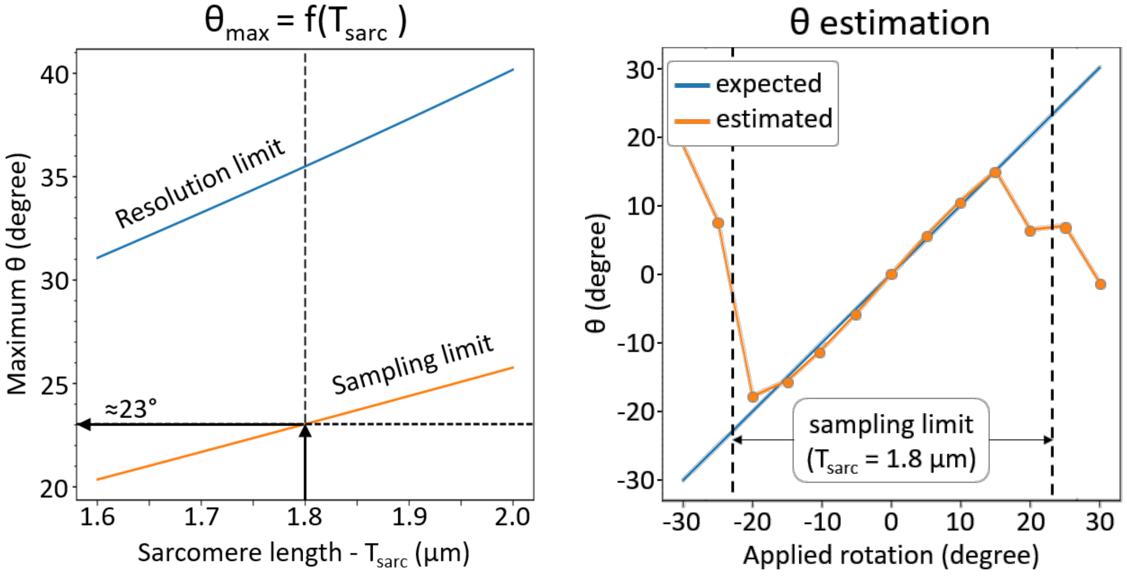

(a) Maximum cell elevation θ. (b) Performance on θ estimation.

Fig. S11: **Out-of-plane angle estimation**. In (a), maximum θ value that allow to resolve (blue) and sample (orange) Z-bands in the image plane is shown as function of sarcomere length T. With an average T of 1.8 µm in the samples, maximum useful value of θ is about 23°. In (b) is shown the average result of the FFT-based orientation analysis applied on 140 portions of tissue selected randomly from TPFM reconstructions of three different septum strips. Each portion has a dimension of 16 µm × 16 µm × 16 µm and it is rotated on θ maintaining $\varphi = 0$. Blue line is the theoretical θ (i.e. the applied rotation), orange spots are θ values estimated with the proposed FFT-based approach. Sampling limit of $\theta = 23°$ is pointed by the dot lines.

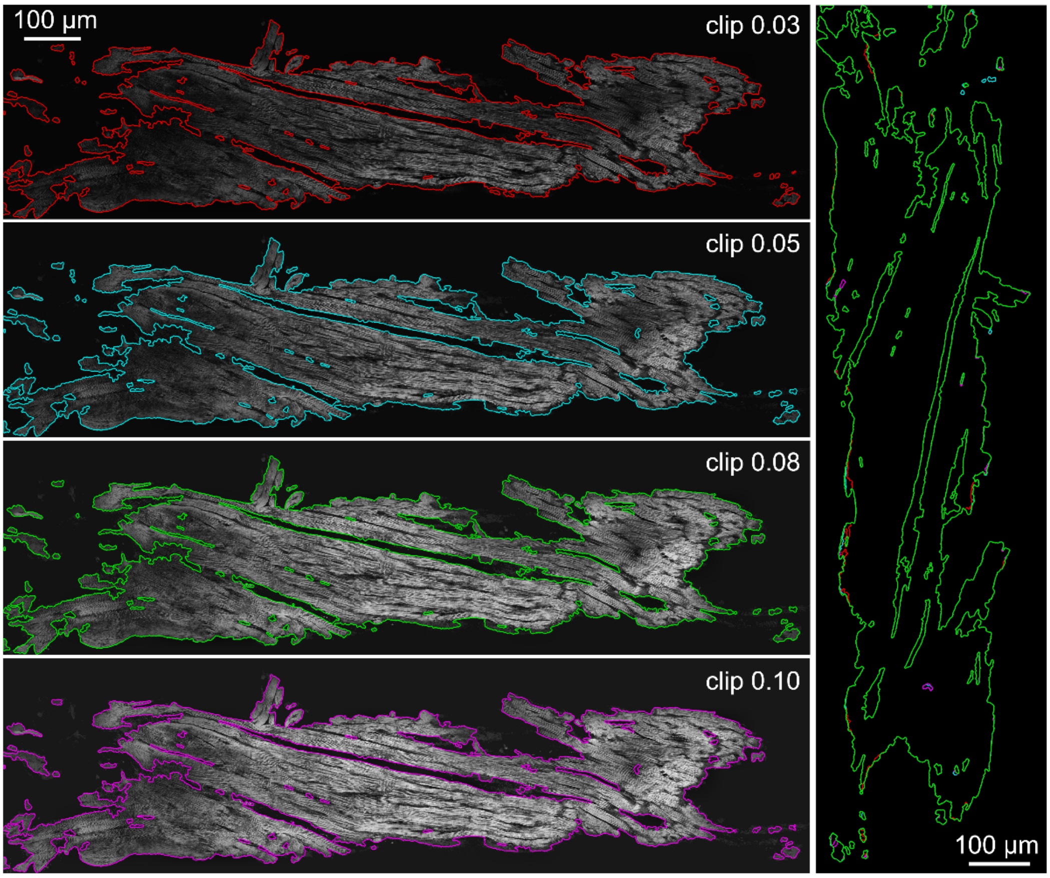

Fig. S12: **Impact of image equalization to the segmentation.** The sensitivity of the K-Means-based segmentation (including 4-clusters quantization and pixel clusters classification) to the image equalization is evaluated on a representative frame of a healthy human septal strip TPFM reconstruction (depth: 150 µm), where α-actinin is stained. Equalization is applied with different clip values (ranging from default value of 0.03 to a maximum of 0.1) and segmentation is performed. Results are shown superimposed to the equalized frame. On the right, results are shown as color layers on a black background to highlight differences.

G. Supplementary tables

Class	Strip	Spatial Resolution	LDD Log-normality	LDD Log-Normal PDF	
		µm	p-value	*µ*	*σ*
IVS	IVS1	32	0,00492	-0,35	0,89
	IVS1	48	0,00930	0,08	0,54
	IVS1	64	0,10722	0,16	0,4
	IVS1	80	0,13269	0,16	0,34
	IVS1	96	-	0,14	0,26
	IVS2	32	0,00000	-0,49	0,82
	IVS2	48	0,00049	-0,14	0,48
	IVS2	64	0,00000	0,01	0,5
	IVS2	80	0,00014	0,04	0,37
	IVS2	96	0,15276	0,1	0,29
	IVS3	32	0,66309	-0,78	0,9
	IVS3	48	0,60685	-0,5	0,61
	IVS3	64	0,01054	-0,35	0,6
	IVS3	80	0,00603	-0,11	0,58
	IVS3	96	0,13237	-0,41	0,22
AF	AF1	32	0,53428	0,94	1,12
	AF1	48	0,00328	1,4	0,59
	AF1	64	0,00228	1,63	0,73
	AF1	80	-	2,04	0,03
	AF1	96	-	2,13	0
	AF2	32	0,00000	1,25	1,01
	AF2	48	0,03465	1,75	0,53
	AF2	64	0,02531	1,82	0,53
	AF2	80	0,36104	2,13	0,29
	AF2	96	-	2,07	0,16

Fig. T1: **Statistical characterization of the Local Disarray Distribution**. Tables show the statistical characterization of the Local Disarray Distributions (LDDs) found in both the cardiac tissue classes: InterVentricular Septum of a healthy donor (IVS), and atrium from a patient suffering from chronic Atrial Fibrillation (AF). Results of each tissue strip (three as control, IVS1-2-3, and two as pathological, AF1-2) and each spatial resolution involved in the local tissue disarray quantification are shown. Log-normality of each set of local disarray values (LDV) is evaluated with D'Agostino-Pearson test, and p-value is reported (if missing, too few LDV are collected across the cytoarchitecture reconstruction). Every LDD is fit with a Log-normal Power Density Function (PDF) defined by (μ, σ).

References

[1] P. Virtanen et al., “SciPy 1.0–Fundamental Algorithms for Scientific Computing in Python,” Nat. Methods, vol. 17, pp. 231–272, 2020. DOI:10.1038/s41592-019-0686-2.

[2] T.E. Oliphant, “A guide to NumPy,” USA: Trelgol Publishing, 2006.

[3] S. van der Walt et al. and the scikit-image contributors, “scikit-image: Image processing in Python,” PeerJ., vol. 2, pp. e453, 2014.

[4] G. Bradski, “The OpenCV Library,” Dr. Dobbs. J. Soft. Tools, 2000.

[5] J.D. Hunter, “Matplotlib: A 2D Graphics Environment,” Comput. Sci. Eng., vol. 9, pp. 90–95, 2007. DOI:10.1109/MCSE.2007.55.

[6] P. Ramachandran and G. Varoquaux, “Mayavi: 3D Visualization of Scientific Data,” Comput. Sci. Eng., vol. 13, no. 2, pp. 40–51, 2011.

[7] C.A. Schneider, W.S. Rasband and K.W. Eliceiri, “NIH Image to ImageJ: 25 years of image analysis,” Nat. Methods, vol. 9, no. 7, pp. 671–675, 2007.

[8] J. Schindelin, I. Arganda-Carreras, E. Frise et al., “Fiji: an open-source platform for biological-image analysis,” Nat. Methods, vol. 9, no. 7, pp. 676–682, 2012. DOI:10.1038/nmeth.2019.
